# Supplementary material for: Incidence and predictors of severe acute malnutrition mortality in children aged 6–59 months admitted at Pawe general hospital, Northwest Ethiopia
Source: PLoS One. 2022 Feb 25;17(2):e0263236. doi: 10.1371/journal.pone.0263236 (PMC8880861; doi:10.1371/journal.pone.0263236)
Supplement: Attachment — Submitted filename: PONE-D-21-11027_R5_editor.pdf [file pone.0263236.s009.pdf]

PLOS ONE

# INCIDENCE AND PREDICTORS OF SEVERE ACUTE MALNUTRITION MORTALITY RATE AMONG CHILDREN AGED 6-59 MONTHS ADMITTED AT PAWE GENERAL HOSPITAL, NORTHWEST ETHIOPIA 2021

--Manuscript Draft--

| | |
|---|---|
| **Manuscript Number:** | PONE-D-21-11027R5 |
| **Article Type:** | Research Article |
| **Full Title:** | INCIDENCE AND PREDICTORS OF SEVERE ACUTE MALNUTRITION MORTALITY RATE AMONG CHILDREN AGED 6-59 MONTHS ADMITTED AT PAWE GENERAL HOSPITAL, NORTHWEST ETHIOPIA 2021 |
| **Short Title:** | INCIDENCE AND PREDICTORS OF SEVERE ACUTE MALNUTRITION MORTALITY RATE AMONG CHILDREN AGED 6-59 MONTH CHILDREN |
| **Corresponding Author:** | Fassikaw Kebede, Bsc, MPH<br>Woldia University, College of Health science school of public health<br>Woldia city, Amhara ETHIOPIA |
| **Keywords:** | Severe acute malnutrition, Predictors of mortality, Under-five-Children, Ethiopia, |
| **Abstract:** | Abstract: Severe acute malnutrition (SAM) is defined by very low weight for height (below -3z scores of the median WHO growth standards), by visible severe wasting, or by the presence of nutritional edema. Mortality magnitudes for under-five children were well documented in Ethiopia, nevertheless lacking information on the predictors when death occurs during inpatient treatment. The aim of understanding to time when under-five children with inpatient SAM die and its predictors could be vital to develop the time-relevant intervention.<br>Methods : A retrospective cohort study was conducted among 568 under-five children from January 1 st , 2015 up to December 31 st , 2019 at Pawe general hospital. Epi Data version 3.2 and Stata (SE) R- 14 software were used for data entry and analysis, respectively. The Kaplan–Meier survival curves and log-rank test were performed to compare significant survival and death experiences among study participants. Bi-variables Cox-regression model was built to select candidate variables at P <0.25. Adjusted hazard ratio (AHR) with a 95% confidence interval (CI) was used to estimate a significant predictor for inpatient SAM mortality at P<0.05<br>Result:- Five-hundred sixty-eight (N=568) admitted SAM cases were included in this study. The mean age of participants was 27.4 (SD± 16.5) months, and the overall incidence density rate (IDR) of inpatient mortality was 16.03 per 100 (95%CI: 13.86; 20.04) person-days risk of observation. Cases at baseline being admitted with vomiting (AHR=5.1; 95%CI: 1.35-21.1, P<0.026), diarrhea (AHR=2.79; 95%CI: 1.46-5.4; P<0.002), nasogastric therapy (NGT) (AHR=3.22; 95%CI; 1.65- 6.26, P<0.001) , anemic (AHR= 1.89; 95% CI: 1.15- 3.2; P<0.012) , and being re-admitted(relapsed) SAM case (AHR=1.7; 95%CI: 1.12 - 2.8, P<0.037) were independent predictors for inpatient SAM mortality.<br>Conclusion:- The overall SAM mortality rate was high among children treated in stabilizing centers as compared with the national standard of protocol(<10%). Cases at baseline admitted with vomiting, diarrhea, NGT, anemic and re-admitted (Relapsed SAM cases) were highly associated with mortality incidence. Health education on early medical seeking behavior for clinicians, caregivers, and adherence to the routine regimens may improve child inpatient treatment survival besides boosting up the strategy to minimize attrition rate after case admission. |
| **Order of Authors:** | Fassikaw Kebede, BSc, MPH epidemiology |
| | Tsehay Kebede |
| | Belete Negese |
| | Atitegeb Abere |
| | Getahun Fentaw |
| **Opposed Reviewers:** | |

Powered by Editorial Manager® and ProduXion Manager® from Aries Systems Corporation

| Response to Reviewers: | Dear editor we would like to thanks to be with this manuscript |
|---|---|
| Additional Information: | |
| **Question** | **Response** |
| **Financial Disclosure**<br><br>Enter a financial disclosure statement that describes the sources of funding for the work included in this submission. Review the submission guidelines for detailed requirements. View published research articles from *PLOS ONE* for specific examples.<br><br>This statement is required for submission and **will appear in the published article** if the submission is accepted. Please make sure it is accurate.<br><br>**Unfunded studies**<br>Enter: *The author(s) received no specific funding for this work.*<br><br>**Funded studies**<br>Enter a statement with the following details:<br>• Initials of the authors who received each award<br>• Grant numbers awarded to each author<br>• The full name of each funder<br>• URL of each funder website<br>• Did the sponsors or funders play any role in the study design, data collection and analysis, decision to publish, or preparation of the manuscript?<br>• **NO** - Include this sentence at the end of your statement: *The funders had no role in study design, data collection and analysis, decision to publish, or preparation of the manuscript.*<br>• **YES** - Specify the role(s) played.<br><br>* typeset | We authors of this research are an Ethiopian citizens |
| **Competing Interests**<br><br>Use the instructions below to enter a competing interest statement for this submission. On behalf of all authors, disclose any competing interests that could be perceived to bias this work—acknowledging all financial support and any other relevant financial or non-financial competing interests. | The authors declare that there are no competing interests. |

| | |
|---|---|
| This statement is required for submission and **will appear in the published article** if the submission is accepted. Please make sure it is accurate and that any funding sources listed in your Funding Information later in the submission form are also declared in your Financial Disclosure statement.<br><br>View published research articles from *PLOS ONE* for specific examples.<br><br>**NO authors have competing interests**<br><br>Enter: *The authors have declared that no competing interests exist.*<br><br>**Authors with competing interests**<br><br>Enter competing interest details beginning with this statement:<br><br>*I have read the journal's policy and the authors of this manuscript have the following competing interests: [insert competing interests here]*<br><br>* typeset | |
| **Ethics Statement**<br><br>Enter an ethics statement for this submission. This statement is required if the study involved:<br><br>• Human participants<br>• Human specimens or tissue<br>• Vertebrate animals or cephalopods<br>• Vertebrate embryos or tissues<br>• Field research<br><br>Write "N/A" if the submission does not require an ethics statement.<br><br>General guidance is provided below. Consult the submission guidelines for detailed instructions. **Make sure that all** | Date 17/ 8/2021<br>To: PLOS ONE<br>Issue: cover letterer<br>Manuscript title:-Incidence and Predictors of Severe Acute Malnutrition Mortality Rate Children Age 6-59 Months Admitted In Stabilizing Center Pawe General Hospital, Northwest Ethiopia<br><br>This is to inform you as members of Ethiopia University the problem is solved based on original studies results and decisions come accordingly by scientific communication. We authors understand to submit our new research on your journals. As you know, Sever acute malnutrition is a silent crisis of killing of in Ethiopia. The problem is a more worse and had devastating impact on under- five children. Besides, to risks of death, there is a growing consensus of once life span long-lasting scary consequences into adulthood for imped school competency and productivity. Therefore, we authors provide entitled incidence rate and predictors of severe acute malnutrition mortality among 6-59 months children admitted in stabilizing center at Pawe general hospitals, northwest, Ethiopia 2020 for publications.<br>Furthermore, the finical support for this research was obtained from Debre Markos university graduate school as MPH student research support fund. The role the funders |

| | |
|---|---|
| **information entered here is included in the Methods section of the manuscript.**<br><br>**Format for specific study types**<br><br>**Human Subject Research (involving human participants and/or tissue)**<br>• Give the name of the institutional review board or ethics committee that approved the study<br>• Include the approval number and/or a statement indicating approval of this research<br>• Indicate the form of consent obtained (written/oral) or the reason that consent was not obtained (e.g. the data were analyzed anonymously)<br><br>**Animal Research (involving vertebrate animals, embryos or tissues)**<br>• Provide the name of the Institutional Animal Care and Use Committee (IACUC) or other relevant ethics board that reviewed the study protocol, and indicate whether they approved this research or granted a formal waiver of ethical approval<br>• Include an approval number if one was obtained<br>• If the study involved *non-human primates*, add *additional details* about animal welfare and steps taken to ameliorate suffering<br>• If anesthesia, euthanasia, or any kind of animal sacrifice is part of the study, include briefly which substances and/or methods were applied<br><br>**Field Research**<br><br>Include the following details if this study involves the collection of plant, animal, or other materials from a natural setting:<br>• Field permit number<br>• Name of the institution or relevant body that granted permission | took in the study, Funders had no role in this study as I am MPH candidate in the abovementioned university funders has no role except giving support for their students on the time of research. In addition, no authors mentioned in this study had received any salary for this particular study. In this study, including me all authors received no specific funding for this work<br>Lastly , we have also added Academic editors (Mr. Bob Taylors) comment with Author response for reviewers issue raised during June re-submission. Thanks for your understand<br>1*Corresponding Author<br>Fassikaw Kebede (BSC, MPH)<br>Lecturer at school of Public health<br>College of Health science Woldia University<br>FK = fassikaw123@gmail.com<br>Institutional email = fassikaw.k@wldu.edu.et<br>Web address URL: http://www.wldu.edu.et |
| **Data Availability**<br><br>Authors are required to make all data underlying the findings described fully available, without restriction, and from the time of publication. PLOS allows rare exceptions to address legal and ethical | Yes - all data are fully available without restriction |

*Powered by Editorial Manager® and ProduXion Manager® from Aries Systems Corporation*

| | |
|---|---|
| concerns. See the PLOS Data Policy and FAQ for detailed information.<br><br>A Data Availability Statement describing where the data can be found is required at submission. Your answers to this question constitute the Data Availability Statement and **will be published in the article**, if accepted.<br><br>**Important:** Stating 'data available on request from the author' is not sufficient. If your data are only available upon request, select 'No' for the first question and explain your exceptional situation in the text box.<br><br>Do the authors confirm that all data underlying the findings described in their manuscript are fully available without restriction? | |
| **Describe where the data may be found in full sentences. If you are copying our sample text, replace any instances of XXX with the appropriate details.**<br><br>• If the data are **held or will be held in a public repository**, include URLs, accession numbers or DOIs. If this information will only be available after acceptance, indicate this by ticking the box below. For example: *All XXX files are available from the XXX database (accession number(s) XXX, XXX.*).<br>• If the data are all contained **within the manuscript and/or Supporting Information files**, enter the following: *All relevant data are within the manuscript and its Supporting Information files.*<br>• If neither of these applies but you are able to provide **details of access elsewhere**, with or without limitations, please do so. For example:<br><br>*Data cannot be shared publicly because of [XXX]. Data are available from the XXX Institutional Data Access / Ethics Committee (contact via XXX) for researchers who meet the criteria for access to confidential data.* | Final Data sets were sent as supporting information (S1 Data.)for journals via STATA |

Powered by Editorial Manager® and ProduXion Manager® from Aries Systems Corporation

| | |
|---|---|
| *The data underlying the results presented in the study are available from (include the name of the third party and contact information or URL).*<br>• This text is appropriate if the data are owned by a third party and authors do not have permission to share the data.<br><br>* typeset | |
| Additional data availability information: | Tick here if the URLs/accession numbers/DOIs will be available only after acceptance of the manuscript for publication so that we can ensure their inclusion before publication.; Tick here if your circumstances are not covered by the questions above and you need the journal’s help to make your data available. |

Powered by Editorial Manager® and ProduXion Manager® from Aries Systems Corporation

**Date 17/ 8/2021**

**To: PLOS ONE**

Issue: cover letterer

**Manuscript title**:-**Incidence and Predictors of Severe Acute Malnutrition Mortality Rate Children Age 6-59 Months Admitted In Stabilizing Center Pawe General Hospital, Northwest Ethiopia**

This is to inform you as members of Ethiopia University the problem is solved based on original studies results and decisions come accordingly by scientific communication. We authors understand to submit our new research on your journals. As you know, Sever acute malnutrition is a silent crisis of killing of in Ethiopia. The problem is a more worse and had devastating impact on under- five children. Besides, to risks of death, there is a growing consensus of once life span long-lasting scary consequences into adulthood for imped school competency and productivity. Therefore, we authors provide entitled incidence rate and predictors of severe acute malnutrition mortality among 6-59 months children admitted in stabilizing center at Pawe general hospitals, northwest, Ethiopia 2020 for publications.

Furthermore, the finical support for this research was obtained from Debre Markos university graduate school as MPH student research support fund. The role the funders took in the study, Funders had no role in this study as I am MPH candidate in the abovementioned university funders has no role except giving support for their students on the time of research. In addition, no authors mentioned in this study had received any salary for this particular study. In this study, including me all authors received no specific funding for this work

Lastly , we have also added Academic editors (Mr. Bob Taylors) comment with Author response for reviewers issue raised during June re-submission. Thanks for your understand

1*Corresponding Author
Fassikaw Kebede (BSC, MPH)
Lecturer at school of Public health
College of Health science Woldia University
FK = fassikaw123@gmail.com
Institutional email = fassikaw.k@wldu.edu.et
Web address URL: http://www.wldu.edu.et

Manuscript

Click here to access/download;Manuscript;Manuscript-R-5-6.docx

# INCIDENCE AND PREDICTORS OF SEVERE ACUTE MALNUTRITION MORTALITY RATE AMONG CHILDREN AGED 6-59 MONTHS ADMITTED AT PAWE GENERAL HOSPITAL, NORTHWEST ETHIOPIA 2021

**Fassikaw Kebede (BSC, MPH) [1*] Tsehay Kebed(BSC,MA)[2] ,Belete Negese(BSc,Msc)[3,] Atitegeb Abera (BSC,MPH)[1], Getahun Fentaw(BSC,MPH)[4]**

[1]Department of Epidemiology and Biostatics, College of Health Science, Woldia University, Ethiopia-2021
[2]Department of Geography and environment study, Faculty of social science, Bahir Dare University, Ethiopia 2021.
[3]Department of Nursing &Midwifery, College of Medicine &Health Science, Debre Birhan University 2021
[4]Department of Nutrition, School of public health, college of health science, Woldia University 2021

**Authors email**

**FK: fassikaw123@gmail.com**
**TK: tsehaynolawit@gmail.com**
**BN: yabebalij@gmail.com**
**AA: atitegebabera@gmail.com**
**GF: gechfentaw1014@gmail.com**

**1*Corresponding Author**
**Fassikaw Kebede (BSC, MPH)**
**Lecturer at school of Public health**
**College of Health science Woldia University**
**FK = fassikaw123@gmail.com**
**Institutional email = fassikaw.k@wldu.edu.et**
**Web address URL: http://www.wldu.edu.et**

**Abstract:** Severe acute malnutrition (SAM) is defined by very low weight for height (below -3z scores of the median WHO growth standards), by visible severe wasting, or by the presence of nutritional edema. Mortality magnitudes for under-five children were well documented in Ethiopia, nevertheless lacking information on the predictors when death occurs during inpatient treatment. The aim of understanding to time when under-five children with inpatient SAM die and its predictors could be vital to develop the time-relevant intervention.

**Methods**: A retrospective cohort study was conducted among 568 under-five children from January 1st, 2015 up to December 31st, 2019 at Pawe general hospital. Epi Data version 3.2 and Stata (SE) R- 14 software were used for data entry and analysis, respectively. The Kaplan–Meier survival curves and log-rank test were performed to compare significant survival and death experiences among study participants. Bi-variables Cox-regression model was built to select candidate variables at $P<0.25$. Adjusted hazard ratio (AHR) with a 95% confidence interval (CI) was used to estimate a significant predictor for inpatient SAM mortality at P<0.05

**Result:-**Five-hundred sixty-eight (N=568) admitted SAM cases were included in this study. The mean age of participants was 27.4 (SD± 16.5) months, and the overall incidence density rate (IDR) of inpatient mortality was 16.03 per 100 (95%CI: 13.86; 20.04) person-days risk of observation. Cases at baseline being admitted with vomiting (AHR=5.1; 95%CI: 1.35-21.1, P<0.026), diarrhea (AHR=2.79; 95%CI: 1.46- 5.4; P<0.002), nasogastric therapy (NGT) (AHR=3.22; 95%CI; 1.65- 6.26, P<0.001) , anemic (AHR= 1.89; 95% CI: 1.15- 3.2; P<0.012) , and being re-admitted(relapsed) SAM case (AHR=1.7; 95%CI: 1.12 - 2.8, P<0.037) were independent predictors for inpatient SAM mortality.

**Conclusion:-**The overall SAM mortality rate was high among children treated in stabilizing centers as compared with the national standard of protocol(<10%). Cases at baseline admitted

with vomiting, diarrhea, NGT, anemic and re-admitted (Relapsed SAM cases) were highly associated with mortality incidence. Health education on early medical seeking behavior for clinicians, caregivers, and adherence to the routine regimens may improve child inpatient treatment survival besides boosting up the strategy to minimize attrition rate after case admission.

**Keywords**: Severe acute malnutrition, Predictors of mortality rate, Children, Ethiopia

## Introduction

Malnutrition remains one of the most common causes of morbidity and mortality in children throughout the world. It has been responsible for directly or indirectly 60%of the 10.9 million death annually among under-five children. Two-third of these deaths occurred during the first year of life **[1-4].** Childhood under-nutrition incorporates a combination of nutrition disorders that include underweight, wasting, stunting, and micronutrient deficiency[5, 6]. Underweight (Weight-For-Age ≤- 3 Z score), is a composite measure of wasting and stunting, while wasting (Weight For Height ≤- 3 Z score) is acute malnutrition due to a recent failure to receive nutrition's and may be affected by recent episodes of diarrhea and other acute illnesses[3, 5]. Although SAM usually affects all segments of a population, infants and young children are most vulnerable as they have higher nutritional requirements for growth and development[7]. It is one of the leading causes of morbidity and mortality among infants and young children all over the world and more frequently in sub-Saharan Africa and South Asia[8]. The peak age for SAM is 6–18 months, which is the time of fast growth and brain development[9]. Globally in 2018, 1 in 12 of the estimated 52 million children under five had SAM[10], and 2.9 million of those children were admitted for inpatient treatment [10, 11]. The Sub-Saharan African countries account for the most proportions of case fatality rate[12]. Despite the availability of

outpatient treatment, the number of children with SAM seeking admission at stabilizing centers (SC) increasing. However,50% of children with SAM die due to inappropriate care after being admitted to stabilizing center [13]. One important reason is poor adherence to SAM therapeutic guidelines [1], and another is the late presentation for treatment centers [14]. Malnutrition in Ethiopia is a long-term silent killing crisis, especially for infants, and children which contribute to an estimated 270,000 deaths each year [15]. According to the Health and Health-Related Indicators (HHRI) of 2014 in Ethiopia, SAM was the third leading cause of mortality and accounted for 8.1%of the death of under-five children [5, 16, 17]. Despite launching the Seqota declaration to end the death of SAM by 100% access to adequate food[18], SAM is still a reason for 20% of pediatric hospital admissions, and 30%of inpatient death [19]. This is the main challenge of successful treatment outcomes in developing countries like Ethiopia and generating a local based data for reason to death is highly important to meet the goal of therapeutic feeding centers. The aim of understanding when inpatient death occurred during treatment and determining its predictors should be vital to develop a time-relevant intervention. Therefore, the aim of this research is to assess incidence and predictors of severe acute malnutrition mortality rate for children admitted in Pawe general hospital, North West Ethiopia 2021.

## Methods

### Study setting, population, and design

A facility-based retrospective cohort study was conducted on 568 under-five children who were admitted for SAM treatment at Pawe General Hospital from January 1st, 2015 to December 31, 2019. Pawe general hospital is located in Metekel Zone Pawe especially Woreda, 565 km in North West direction of Addis Ababa, the capital city of Ethiopia [20, 21]. According to the 2019 national population projection, this region has an estimated 1.21 million population[22].

The Pediatric ward is among the five inpatient departments found in Pawe general hospital with 252 beds for inpatient treatment and a separate stabilizing center for children with SAM. Ethiopia adopted a set of world health organizations for severe malnutrition treatment guidelines of under-five children using three phases; the first phase (phase I), the transition phase, and phase II. In all phases admitted children are treated empirically for infections, hypoglycemia, and hypothermia and resuscitated to restore electrolyte balance. The hospital provides service for total catchment populations and nearby regions like Amhara both northeast and Westside. Following the time of SAM admission, from 1st January 2015, up to 31st December 2019, there were a total of 578 Under-five children had been registered for SAM inpatient treatments.

**Sample size determination**

We determined the sample size for this study both by single population proportion and survival analysis formula using the following assumption; n =(Za/2)2P (1-P)/d2 and considering a 95% confidence level (Z a/2 = 1.96), the margin of error 5%, overall mortality rate 46%[6], and 15 % addition for incomplete data and yields 454. Whereas, the sample size for the second objective was achieved using STATA/SE version-14 by considering two-sided significance level ($\alpha$ = 5%), $Za_{1/2}$ = Z value at 95% confidence interval = 1.96, Power ($Z_B$)=80%, and P = % cumulative occurrence of death rate, 1.65 HR.

The final *s*ample size $(n) = \frac{Event}{P\,(Event)} = \frac{(Z_{a/2}+Z_B)2}{\theta^2 p\,(1-p)} = \frac{(za/2+ZB)}{P\,(1-p)(lnHR)2}$ [23]

$\theta$ = ln(HR)

HR= $e^{\theta}$

e = event

P (e) = probability of event

P = Mortality rate 46%from [6].

HR= hazard ratio (AHR)0.6 from [6].

By inserting all parameters into the STATA (SE)\14 gives 448. However, the five years multi-chart recoded reviewed only 578 records were there and those are manageable and included all for final analysis.

**Outcome ascertainment**

In this study, the outcome of interest was inpatient death due to SAM, and death was defined as following after admission to report of death during treatment observation. Variables like age, sex, residence, vaccination status, vomiting, breastfeeding, NGT, medication administration, admission types, malnutrition types, and mid-upper are circumference status, medical comorbidity, etc., were considered as independent variables for the development of the checklist.

**Operational words**

**Admission criteria:-**According to WHO management of SAM a children who are 6–59 months of age, Weight-for-height ≤–3 Z-score, or mid-upper-arm circumference <115 mm, or presence of bilateral edema, and failed appetite test should be admitted for inpatient care[24].

**Discharged /declared Cured:** According to WHO management of SAM a children who are 6–59 months of age, during inpatient treatment declared as cured when weight-for-height/length is ≥–2 Z-score and they have had no edema for at least 2 weeks, or. The mid-upper-arm circumference is >115 mm and they may have had no edema for at least 2 weeks or [7].

**Defaulted/Abscond/Lost from following up:-**when severe acute malnutrition diagnosed &admitted children absent from stabilizing center for consecutive 2 days after started treatment[5].

**Anemia in children:-** was defined and classified based on the mean amount of red blood cell or Hgb levels mainly classified into two ways; Anemia(Hgb) <11 g/dL and No anemia(Hgb) ≥11 g/dL[25].

**Data collection instrument and quality controls**

A structured English version checklist was developed and used for data extraction from admitted children's medical records on the Federal Ministry of Health (FMOH) severe acute malnutrition [7]. One day of training was given for three diploma nurses and One BSC public health officer for data collection tools, data extraction system, and the objectives of the study. To assure the quality of data, the data collection checklist was 5% of the total sample size was pretested. After the pretest, necessary modification of the data collection tool was incorporated. Strict follow-up and supervision were carried out during data collection by principal investigators and feedback was given daily. The collected data were reviewed &checked for completeness before data entry.

**Data processing and analysis**

The collected data were entered using Ep-iData version 4.2 statistical software and exported to STATA (SE) R-14 version statistical software for further analysis. The proportional hazard assumption was checked for each variable and no variable was found with Schoenfeld residual test <0.05. The Kaplan–Meier survival curves and log-rank test were performed to compare significant experience among survival /death study participants. Variables with P-value < 0.25 in bi-variable Cox regression analysis were included into multivariable Cox regression model. Finally, a variable with an adjusted hazard ratio (AHR) it 95% confidence interval (CI) at P-value <0.05 were considered as significant predictors for inpatient mortality admitted children. The model fitness was checked using the Nelson- Aalen cumulative hazard rate relative to Cox-Snell residuals.

**Ethical statement and consent to participation**

Ethical clearance was obtained from the institute of ethical review committee from Debre Markos University, College of Health Sciences (Ref. No: HSC/984/16/12). A formal letter was

submitted to Pawe General Hospital for permission to be done entitled of the research articles, *Incidence, and predictors of severe acute malnutrition mortality rate children 6-59 months admitted in stabilizing center at Pawe general hospital, North West (2015-2019)***.** Finally**,** Pawe hospital rechecked for ethical compatibility and permitted data access. According to national research ethical review guideline:-https://www.ccghr.ca/wp-content/uploads/2013/11/national-research-ethics-review-guidline[26] if the study was conducted through individual file records reviewed, no need for consent from the mothers or caregivers of the study subject. However, all data set were kept confidential anonymously. The institution of the ethical review committee of Debre Marko's University was also waived consent from the caregiver for all secondary data.

## Result:

### Baseline Socio-demographic and clinical characteristics

After excluding 10(1.74%) individual files due to incompleteness, we reviewed 568 files of SAM cases registered for treatment from 1$^{st}$ January 2015 to December 31 /2020. Out of the included participants, slightly more than half, 324(57.04%) of participant children were females, and the majority, 356 (62.68 %) them were found in the age group 6-24 months. The mean age of the participants was 26.28 (SD= ±16.04) months. Seventy-eight percent of cases were from rural residents, and more than half 361(63.56%) of them were on breastfeeding. Moreover, 169(29.86%) &119(21.02%) SAM cases were admitted during 2019&2015 years, respectively **(Figure 1)**.

### Descriptive result of severe acute Malnutrition

Nearly four in five, 457(80.46%) of the participant cases were new severe acute malnu8tration cases of the total 568SAM cases 318(55.9%) were admitted due to wasting; while the remaining 153(26.94), and 97(17.08) were due to Marasmus-Kwashiorkor and edematous (Kwashiorkor),

respectively. Nearly seventeen percent (68.3% of wasting cases were observed in the 6-24 months age groups. Of the total of 97(17.08) edematous cases, nearly half (48.9%) of them were cured (**Table1).**

**Co-morbidity and Antibiotics**

Moreover, 296(52.18%) of children had pneumonia during admission, while nearly two in five 217 (38.2%) of admitted cases had skin dermatitis. Of all 568 participants, 307 (54.35%) had multiple (more than two) comorbidity during admission, for instance, SAM with diarrhea (11.17%) plus pneumonia (10.55%), and SAM with anemia (19.3%) plus vomiting (14.8%). More than half of 53.32% of the admitted cases had altered body temperature (≥37.5 °C). In addition, 470 (82.75%) and 242 (42.61%) children received vitamin A and deworming syrup during inpatient care, while only 22.8% of children received intravenous fluid **(Table 2).**

**Treatment outcomes of the stabilizing centers**

At the end of this study period, 326 (57.39%) admitted under-five children had been cured, while the remaining 106 (18.66 %) lost from follow-up, 46 (8.10%) transferred out, and the remaining 91 (16.03%) children died. During in-patient treatment of cases, the majority of deaths, 38 (41.11%), were reported during Phase I within 24-144 hours after admission whilst 124 (38.6%) of SAM children were cured in phase II **(Table3).**

**The incidence rate of SAM mortality**

The study participants were followed for 5108 Person per Month (PMOS) risk observation. The overall incidence of the mortality rate was 16.03 per 100 (95%CI: 13.86; 20.04) person-day observations**.** The median duration from admission to death was 13 (IQR=±8) days. The proportion of new death during inpatient treatment at the end of 1$^{st}$ week, 2$^{nd}$ week, 3$^{rd}$ week, and

end of follow up were, 28/91(30.76%),26/91(28.57%), 16/91(17.5%), 18/91(19.78%), and 3/91(3.3%), respectively.

**Log rank estimate of mortality and death Hazard**

The log-rank test estimate revealed that the mortality among SAM admitted children were significantly varied among the covariates. The Kaplan Meier survival curve together with the log-rank test shows the effect of each variable on mortality of SAM admitted children on different covariates (**Table4).**

Baseline categorical variables like anemia, vomiting, diarrhea, and NGT during admission had significant times of survival differences when compared to their counterpart. For instance, the mean survival time for those who had anemia during admission was 18.6 (95%CI; 17.38, 19.9) days and it was 27.5 95%CI; 25.8 29.1) days for those who had no anemia during SAM admission (P-value, <0.0001). The mean survival time for newly admitted SAM children was 24.4(95%CI; 22.9, 25.7) days, while it was 6.4 (95%CI; 4.6, 8.2) days (p-value<0.0001) **(Figure 2-8).**

**Predictors of SAM Mortality**

During bi-variable Cox regression analysis was running 17 variables were run and subsequently, 11 variables were transferred into multivariable Cox regression by p-value <0.25 criteria of regression. After adjustment of potential confounding, five variables found significant predictors for SAM inpatient mortality. The risks of death for children admitted with NGT was three times (AHR=3.22; 95%CI; 1.65- 6.26, P<0.001) increased as compared with children without NGT. Death risk for Re-admitted SAM diagnosed cases were nearly two-times increased as compared with new admission case (AHR=1.7; 95%CI: 1.03—2.8, P<0.037). Hazards of death for children having vomiting during admission was five times increased as compared with no

vomiting case at admission nearly five times (AHR=5.1; 95%CI:1.35—21.1, P<0.026). Likewise, the death hazard for children admitted with anemia was twice increased as compared with children with no anemia (AHR= 1.89; 95% CI: 1.15—3.2; P<0.012). The risk of death for SAM diagnosed and admitted children with diarrhea was nearly three times higher as compared with no diarrhea at admission (AHR=2.79;95%CI:1.46--5.4 ;P<0.002) (**Table 5).**

## Discussion

This study was primarily intended to estimate the incidence of inpatient SAM mortality rate and its potential predictors after admitted children in stabilizing centers from 1st January 2015 up to December 31, 2019. These studies have indicated that at the end of follow up 326 (57.39%) SAM cases had been cured, while the remaining 106 (18.66 %) lost from follow-up, 46 (8.10%) transferred out, and 91(16.03%) cases have died. The overall incidence density rate (IDR) of mortality was 16.03 per 100 (95%CI: 13.86; 20.04) person-days risks of observations. This was higher than the national SPHERE reference (<10%) [12] and might be due to delayed presentation to the health institution (SC), early discontinuation of treatment due to insufficient financial means admissions [5], and poor adherence to WHO SAM guidelines [1, 27]. However, our finding is not consistent and higher than reported in Mekele hospital 3.8%[3], Tigray general hospitals 6.65%[3], Dilla referral hospital 7.57%[1], Bahir-dare referral hospital 7.7%[28], Southern hospital 10.8%[29]. The difference might be due to all these are specialized universities and referral hospitals, which provide excellent and comprehensive treatment care, unlike the counterpart of the pastoralist community in our studied hospital. In sum, the median time to death was reported as 13 (IQR=8) days. This is not consistent and higher than reported in Gondere hospital 12 days[5], but lower than findings in southern hospitals 17 days [29], Mekele referral hospital 41.2 days[3], and Shebedino hospital 36 days [2]. Unfortunately, the economic

constraints of care supporters to buy additional drugs and food exposed for early loss from inpatient care. This is a real scenario in our study setup; more than 18.66%of admitted cases were lost from follow-up. Regarding predictors for inpatient mortality, the hazard of death for anemic children during admission was two-fold increased as compared with the counter group. This is comparable and consistent with the finding in Gonder referral hospitals [5], and Nekemte Referral Hospital [30]. In contrast to our study reported, the study result has no association role for anemia prevention by oral medications( e.g., vitamin A= 82.75% and folic acid =76.94 %), but a study result in NRH [30] revealed a significant association dearth of deworming (P=0.031) prevention versus anemia incidence (0.042) [30]. A child may experience more than one episode of SAM, depending on the improvement of the underlying factors during inpatient treatment [31] similar to this, our research finding revealed SAM relapses have a two-fold hazard of death. In line with the study finding in Lusaka, Zambia [32], Gonder referral hospital [33], Southern Ethiopia hospital [2], and Irena [32], SAM children admitted with diarrhea had an increased risk of death, consistent with our two-fold increase in death, which may be due to combination of fluid and electrolyte loss and possible hypovolemic shock. Moreover, the hazard of death among children with vomiting at admission is three times increased as compared with the counter group. This is consistent with the study finding at North Gonder hospital [33]. Both vomiting and diarrhea are associated with shifting of physiological fluids homeostatic and easily loss of continuity for children whatever medication administration orally. This could be due to the shrinking of the intracellular potassium pumping balance of the body homeostatic. On the other hand, the hazards of death for children at baseline admitted with nasal-gastric therapy (NGT) were two times increased as compared with no NGT admissions. Unlike in our research reported, there is no positive association between admitted SAM cases with shock, vomiting, and poor

adherence, however, study finding in Dilla referral hospital [13], and Gonder referral Hospital [5] had significant associations for the cause of inpatient mortality of Vomiting (P<0.001), altered breathing(P<0.001), and admission with NGT (P<0.038).

## Limitations

The possible limitation of this study is that since it is a record review, it failed to consider a broad range of factors like some biochemical indices and the socioeconomic status of caregivers. Therefore, the interpretation and application of the finding for decision and policy direction should account for these inherent limitations of the study.

**Conclusion:** The overall SAM mortality rate was high among children treated in stabilizing centers as compared with the national standard of protocol. Cases at baseline admitted with vomiting, diarrhea, NGT, and re-admitted had highly associated for mortality incidence. Health education on early medical seeking behavior and adherence on the routine regimens may improve child survival besides to boost up a strategy to minimize attrition after admission

### Abbreviation

AHR, adjusted hazard ratio; CHR, crude hazard ratio; CI, confidence interval; FMOH, Ethiopian Federal Ministry of Health; MUAC, mid-upper arm circumference; SC, stabilizing center; SAM; severe acute malnutrition; NGT, nasal gastric intubation for feeding; WFH, weight for height; SD, standard deviation.

### Acknowledgment

The authors would like to acknowledge Pawe General Hospital's administrative staff.

### Supporting information

All relevant data are within the paper and its **Supporting Information** **files.**

### Conflict of interest

The authors declare that there are no conflicts of interest for this study

**Funding**

There is no especial grant of funding for this research from any organization

**Author's contribution**

Conceptualization; Fassikaw Kebede , Tsehay kebede ,

Data cu ration: Fassikaw Kebede, Tsehay kebede,

Formal analysis; Fassikaw Kebede, Tsehay kebede

Methodology; Fassikaw Kebede,

Software; Fassikaw Kebede, Belete Negese, Tsehay kebede

Supervision; Fassikaw Kebede ,Getahun Fentaw

Writing – original draft: Fassikaw Kebede, Getahun Fentaw

Writing – review & editing: Fassikaw Kebede, Tsehay kebede, Atitegeb Abera ,

## Reference

1. Tadele Girum, M.K., Befikadu Tariku, Incidence and Predictors of Mortality among Severe Acute Malnourished Under Five Children Admitted to Dilla University Referal Hospital: A Retrospective Longitudinal Study. Journal of Biology, Agriculture and Healthcare, 2016. **Vol.6, No.13, 2016**(ISSN 2224-3208 (Paper)).

2. Genene Teshome1, T.B.a.S.G., Time-to-recovery from severe acute malnutrition in children 6–59 months of age enrolled in the outpatient treatment program in Shebedino, Southern Ethiopia: a prospective cohort study. BMC Pediatrics, 2019. **13**(https://doi.org/10.1186/s12887-019-1407-9).

3. Gebremicael Guesh1, G.D., Mebrahtu Abay3, Berhe Beyene3, Ermyas Brhane3* and Kalayu Brhane4, Survival status and predictors of mortality among children with severe acute malnutrition admitted to general hospitals of Tigray, North Ethiopia: a retrospective cohort study. BMC Research Notes, 2018. **https://doi.org/10.1186/s13104-018-3937-x**.

4. World Health Organization :Key malnutrition global report  Malnutrition report, 2018.

5. Fasil Wagner, Mengistu Mekonnen, and Amanuel Alemu Abajobir, Predictors of mortality among under-five children with severe acute malnutrition, Northwest Ethiopia: an institution based retrospective cohort study. Archives of Public Health 2018. **(2018)** (https://doi.org/10.1186/s13690-018-0309-x).

6. Tendai Munthali, C.J., Lungowe Sitali, Rosalia Dambe, and Charles Michelo, Mortality and morbidity patterns in under-five children with severe acute malnutrition (SAM) in

Zambia: a five-year retrospective review of hospital-based records archives of public health (2015),. **DOI 10.1186/s13690-015-0072-1**.

7. FMOH, Protocol for the management of severe acute malnutrition children in ethiopian context. SAM management manual, 2007 Accesed june 2021.

8. Global_Nutrition_Report shining a light to spur action on nutrition, 2018 Report Acesed 10 September 2021 .

9. Wagner Tesfay1, M.A., Solomon Hintsa Tekia Zafu, Length of stay to recover from severe acute malnutrition and associated factors among under-five years children admitted to public hospitals in Aksum, Ethiopia. PLOS ONE, 2020. **e0238311.https://doi.org/10.1371/journal.pone.0238311**.

10. UNICEF and World Health Organizarion, key finding &levels, and trends in child malnutrition. 2018

11. DOCUMENT, U.P.G., management of severe acute malnutrition in children: working towards results at scale. Maule 2014.

12. Australian Agencyfor International Development: SPHERE project in humanitarian charter and minimum standards in humanitarian response. The guideline, 2011. **Third edition** (ISBN 978-1-908176-00-4): p. 402.

13. Girum, T., et al., Survival status and predictors of mortality among severely acutely malnourished children <5 years of age admitted to stabilization centers in Gedeo Zone: a retrospective cohort study. Ther Clin Risk Manag, 2017. **13**: p. 101-110.

14. Banga, D., et al., Comorbidities and Factors Associated with Mortality among Children under Five Years Admitted with Severe Acute Malnutrition in the Nutritional Unit of Jinja Regional Referral Hospital, Eastern Uganda. Int J Pediatr, 2020. **2020**: p. 7809412.

15. Habtemu Jarso, Abdul halik W orkicho, Fisehaye Alemseged: Survival status and predictors of mortality in severely malnourished children admitted to Jimma University Specialized Hospital from 2010 to 2012, Jimma, Ethiopia: a retrospective longitudinal study. BMC Pediatrics 2015. **15**(DOI 10.1186/s12887-015-0398-4).
16. ETHIOPIA,Fedral demogcratic republic of Ethiopia Seqota Declaration Innovation Phase Investment Plan 2018 - 2020. MANUAL, 2018.
17. Nigatu M.a.T.H.: Modeling trends of health and health-related indicators in Ethiopia (1995-2008): a time-series study. Health Research Policy and Systems, Accepted: 13 December 2009. **7:29 doi:10.1186/1478-4505-7-29**.
18. CoHA, Social and Economic Impact of Child Undernutrition in Egypt, Ethiopia, Swaziland , and Uganda. The report, 2010.
19. Fasil Wagnew1 Mengistu Mekonnen, and Amanuel Alemu Abajobir3, Predictors of mortality among under-five children with severe acute malnutrition, Northwest Ethiopia: an institution based retrospective cohort study. Archives of Public Health (2018) 76:64, 2018. **https://doi.org/10.1186/s13690-018-0309-x**.
20. Fassikaw Kebede1, Nemera Eticha2, Belete Negese3, Mastewal Giza1, Tadesse Tolossa4 and Bizuneh Wakuma5, Predictors for a Cure Rate of Severe Acute Malnutrition 6-59 Month Children in Stabilizing Center at Pawe General Hospital, Northwest Ethiopia: Retrospective Cohort Study. International Journal of Child Health and Nutrition, 2021, 10, 34-43, 2021. **10**(https://doi.org/10.6000/1929-4247.2021.10.01.5): p. 10.
21. Fassikaw Kebede, B.K., 2 Tsehay Kebede,3 and Melaku Agmasu4, Effect of Isoniazid Preventive Therapy on the Incidence of Tuberculosis among Seropositive Children Attending HIV/AIDS Care in Two General Hospitals, Northwest Ethiopia, 2021.

Hindawi Journal of Tropical Medicine Volume 2021, Article ID 9996953, 9 pages https://doi.org/10.1155/2021/9996953, 2021.

22. UNICEF, Situation Analysis of Children and Women: Benishangul-Gumuz Region. REPORT 2019.
23. Moore, D.F., Dirk F. Moore: Applied Survival Analysis Using R. Piscataway. , NJ, USA: Springer Nature; 2015., 2015.
24. world health organization(WHO), Updates on the management of severe acute malnutrition in infants and children Geneva: World Health Organization; 2013. Guideline, 2013.

25. Deepak Dwivedi, V.S.J.S. and Sangita Sharma, Study of Anaemia in Children with Severe Acute Malnutrition. J. Nepal Paediatr. Soc., 2017. **/Vol 37/Issue 3**(DOI: http://dx.doi.org/10.3126/jnps.v37i3.18480).
26. EthiopianMinistry of Science and Technology, Ethiopian National-research-ethics-review-guideline. Book, published 2014, accessed 2021.
27. Tadele Girum1, E.M.A.W., Comparative Analysis of the Survival Status and Treatment Outcome of Under-five Children Admitted with Severe Acute Malnutrition Among Hospital-based and Health Center-Based Stabilization Centers, South Ethiopia. The Open Public Health Journal, 2018. **11**(DOI: 10.2174/1874944501811010209): p. 10.
28. Hanna Demelash Desyibelew1, A.F., Haile Woldie3, Recovery rate and associated factors of children age 6 to 59 months admitted with severe acute malnutrition at inpatient unit of Bahir Dar Felege Hiwot Referral hospital therapeutic feeding unite, northwest Ethiopia. PLOS ONE, 2017. **| DOI:10.1371/journal.pone.0171020 F**.

29. Fikrie, A., A. Alemayehu, and S. Gebremedhin, Treatment outcomes and factors affecting time-to-recovery from severe acute malnutrition in 6–59 months old children admitted to a stabilization center in Southern Ethiopia: A retrospective cohort study. Italian Journal of Pediatrics, 2019. **45**(1).

30. Muluken Berhanu Mena, M.G.D., 2 and Bruke Berhanu Billoro3, Treatment Outcome of Severe Acute Malnutrition and Its Determinants among Pediatric Patients in West Ethiopia. Hindawi Publishing International Journal of Pediatrics, 2018, (Article ID 8686501, 7 pages).

31. Abera Lambebo,T.D Tefera Belachew:Frequency of relapse for severe acute malnutrition and associated factors among under-five children admitted to health facilities in Hadiya Zone, South Ethiopia. PLOS ONE 2021(https://doi.org/10.1371/journal.pone.0249232

32. Abel H Irena : Diarrhea is a major killer of Children with Severe Acute Malnutrition Admitted to Inpatient setup in Lusaka, Zambia. BMC Nutrition Journal 2011, 10:110, Iren Nutrition Journal 2011, 10:110 http://www.nutritionj.com/content/10/1/110).

33. Mamo, W.N., et al., Time to recovery and determinants of severe acute malnutrition among 6–59 months children treated at an outpatient therapeutic program in North Gondar zone, Northwest Ethiopia: a prospective follow-up study. Italian Journal of Pediatrics, 2019. **45**(1).

**Table1:** Socio-demographic and clinical characteristics of SAM admitted children in Pawe General Hospital, North West Ethiopia (2015-2019)(n=568).

| Variables | Characters | Frequency % |
|---|---|---|
| Sex | Male | 244(42.96) |
| | Female | 324 (57.2) |
| Residence | Urban | 126(22.18) |
| | Rural | 442(77.82) |
| Age | Between 6-24 month | 355(62.68) |
| | Between 24-48 month | 160(28.17) |
| | Above ≥48 month | 53(9.15) |
| SAM types | Wasting(Marasmic) | 318(55.99) |
| | Marasmus Kwashiorkor | 153(26.94) |
| | Kwashiorkor( Edematous ) | 97(17.08) |
| Skin dermatitis | Absent | 353(61.8) |
| | Present | 217(38.2) |
| Vomiting | Absent | 281(47.89) |
| | Present | 296(52.13) |
| Altered body temperatures $\geq 37.5C^0$ | Absent | 265(46.65) |
| | Present | 303(53.35) |
| Diarrheal | Absent<br>Present | 257(45.2)<br>311(54.75) |
| Pneumonia | Absent | 272(47.89) |
| | Present | 296(52.11) |
| Anemia | Absent | 399(70.75) |
| | Present | 169(29.75) |
| Breastfeeding status | No breastfeed | 207(36.4) |
| | Yes breastfeed | 361(63.56) |
| Admission type | New admission | 457(80.46) |
| | Re-admission | 111(19.54) |
| Nasogastric tube during admission | Present | 243(41.02) |
| | Absent | 325(58.98) |
| Vitamin A supplementation | Given | 470 (82.7) |
| | Not given | 98( 17.25) |
| Folic acid | Given | 406 ( 71.48 ) |
| | Not given | 162 (28.52 ) |
| Blood transfusion during admission | Given | 123 (21.65) |

| | | |
|---|---|---|
| | Not given | 445 ( 78.35) |

**Table2**:-Performance indicator of stabilizing center with compared *Sphere reference* for Incidence rate and predictors of Under-five SAM children in Pawe General Hospital (n=568).

| **Performance indicator** | **Five years Performance of Stabilizing center** | **SPHERE project reference value** | | |
|---|---|---|---|---|
| | | Overall | Acceptable | Alarming |
| Treatment cure rate | 57.38% | 77.9% | >75% | <50% |
| Incidence of death rate(IDR) | 16.7% | 12.3% | <15% | >25% |
| lost from follow up | 18.36% | 5.5% | <10% | >15% |
| Transfer out | 8.7% | 4.8% | ____ | ____ |

Table3: Proportions of SAM treatment outcome by phase after being admitted to stabilizing center in Pawe general Hospital (2015-2019 (n=568).

| Indicators | **Phase 1** | **Transition phase** | **Phase 2** | **Treatment outcome by phases(N=568)** |
|---|---|---|---|---|
| **Death rate** | **38(41.11)** | **24(26.4)** | **29(31.50)** | **91** |
| **Treatment cure rate** | **97 (29.56)** | **105(32.39)** | **124(38.60)** | **326** |
| **Lost follow up** | **9(8.50)** | **34 (32.81)** | **63 (60.0)** | **106** |
| **transferred out** | **3(6.52)** | **5(10.8)** | **38(82.6)** | **46** |

Table-4: Log-rank estimate selected variable for SAM admitted children in Pawe General Hospitals in North West Ethiopia, 2021.

| ***Categories*** | **log rank test estimated** | **P - Value** |
|---|---|---|
| Age(years) | $X^2 = 64.36$ | **P-value = 0.053** |
| Sex(Male/ female) | $X^2 = 2.71$ | **P -value = 0.099** |
| Residence(Urban /rural ) | $X^2 = 3.23$ | **P -value = 0.63** |
| Admission types(New/ Re-admission) | $X^2 = 37.02$ | **P - value = 0.001** |
| Had NGT during admission (present /absent ) | $X^2 = 50.22$ | **P -value = 0.001** |
| Measles vaccine (Taking /Missed) | $X^2 = 10.98$ | **P -value = 0.009** |
| Parenteral Antibiotics (Given/ Not given) | $X^2 = 9.16$ | **P -value = 0.003** |
| Vitamin A supplementation (Given/Not Given ) | $X^2 = 1.82$ | **P -value =1.7891** |
| Anemia during admission (Present /Absent) | $X^2 = 46.89$ | **P -value =0.001** |
| Vomiting during admission (Present /Absent ) | $X^2 = 23.02$ | ***P -value =0.001*** |
| Diarrhea during admission (Present/Absent) | $X^2 = 38.9$ | ***P -value =0.001*** |
| Comorbidity during admission (Present /Absent) | $X^2 = 2.15$ | ***P -value =0.645*** |

**Table 5: Bi-variable and multivariable Cox regression for predictors of SAM mortality among children to Pawe general hospital since 2015-2019 (n=568), North West Ethiopia.**

| Covariate | Categories | Survival status | | | | |
|---|---|---|---|---|---|---|
| | | Death | Censored | CHR(95%CI) | AHR(95%CI) | P-value |
| **Age of children** | 6-24 Month | 68 | 186 | 1 | | |
| | 24-48 month | 22 | 145 | 0.9(0.57--1.4) | 1.2(0.73-- 2.01) | 0.443 |
| | ≥48 month | 1 | 46 | 0.61(0.59 -4.4) | 2.1 (0.82- -3.9) | 0.27 |
| **Sex** | Male | 30 | 214 | 1.13(0.89--1.43) | 0.96 (0.61 --1.53) | 0.859 |
| | Female | 61 | 263 | 1 | 1 | |
| **Admission types** | **New** | 67 | 183 | 1 | 1 | |
| | **Re-admission** | 24 | 294 | 1.7(2.4 --- 6.1) | 1.7 (1.03—2.8) | 0.037* |
| **NGT during Admission** | **Yes** | 79 | 164 | 2.3(2.3--6.99) | 3.22(1.65—6.26) | 0.001* |
| | **No** | 12 | 313 | 1 | 1 | |
| **Vomiting** | **Yes** | 88 | 208 | 2.34(1.28 ---4.3) | 5.1(1.35---21.2) | 0.026* |
| | **No** | 3 | 269 | 1 | 1 | |
| **Anemia** | **Present** | 64 | 105 | 4.2(2.6---3.67) | 1.89(1.15–3.12) | 0.012* |
| | **Absent** | 27 | 372 | 1 | 1 | |
| **Diarrhea** | **Yes** | 79 | 232 | 5.6(2.99—10.6) | 2.79(1.46—5.4) | 0.002* |
| | **No** | 12 | 245 | 1 | 1 | |

Click here to access/download;Figure;Figure 2.tif

**Figure 2:** Overall Kaplan Meier their estimated survival of SAM admitted children in Pawe general hospitals since 2015-2019 (N=568).

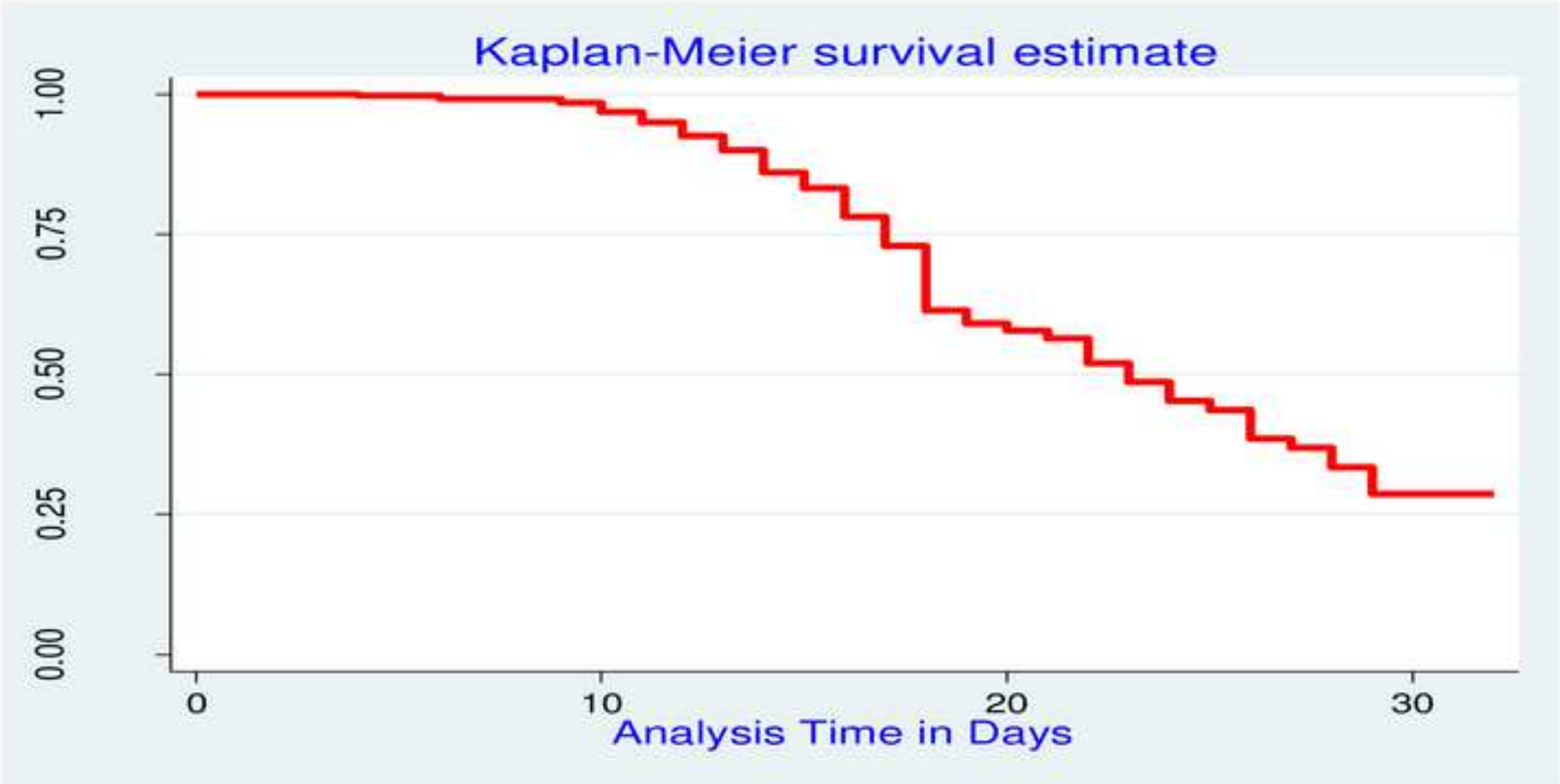

Click here to access/download;Figure;Figure 3.tif

**Figure**:3 Difference in Hazard of death based on Vomiting status of SAM admitted children in Pawe general hospital since 2015-2019 (N=568).

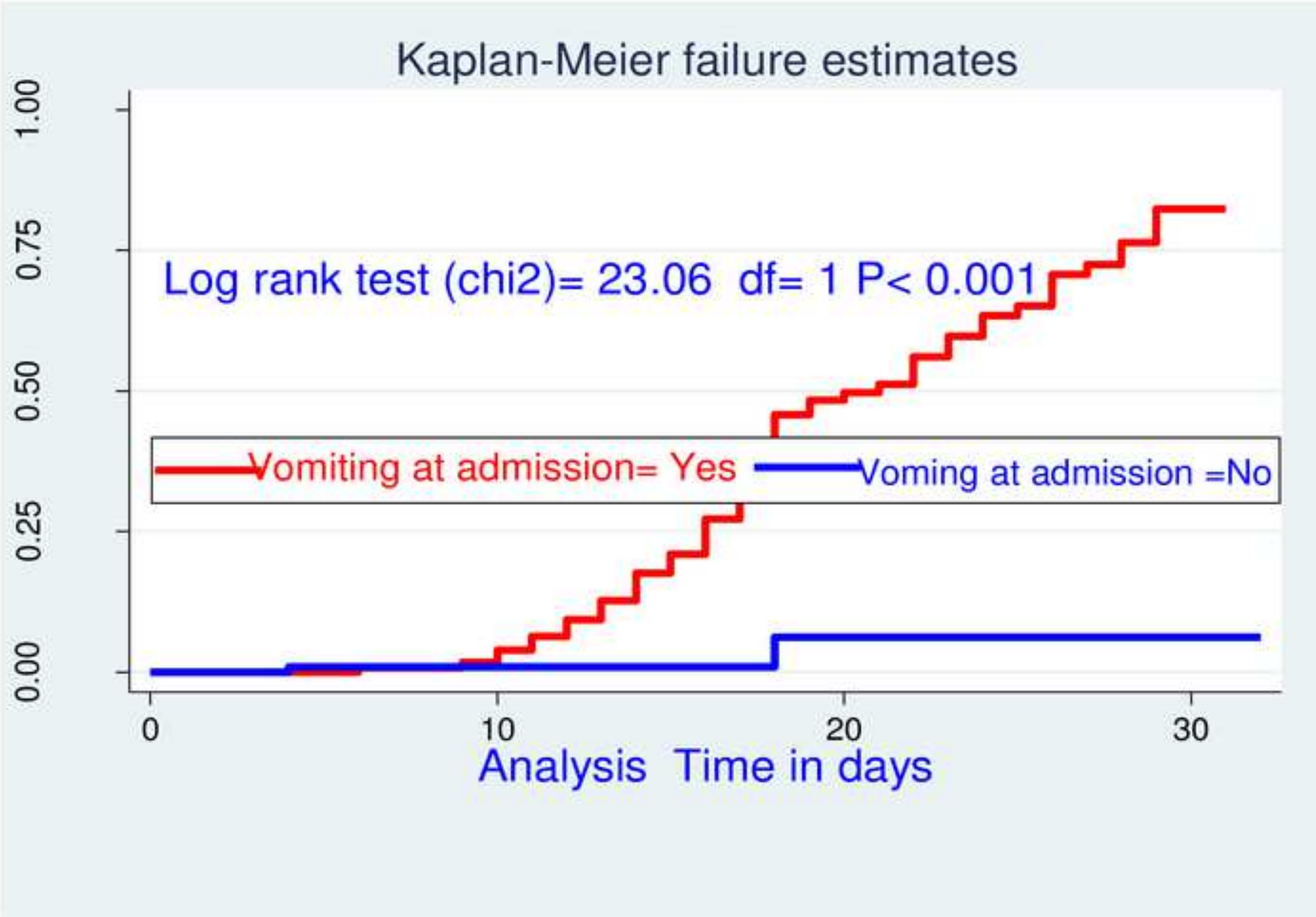

Click here to access/download;Figure;Figure 6.tif

**Figure:6** Difference in Hazard of death based on nasogastric therapy status during SAM admitted children in Pawe general hospital since 2015-2019 (N=568).

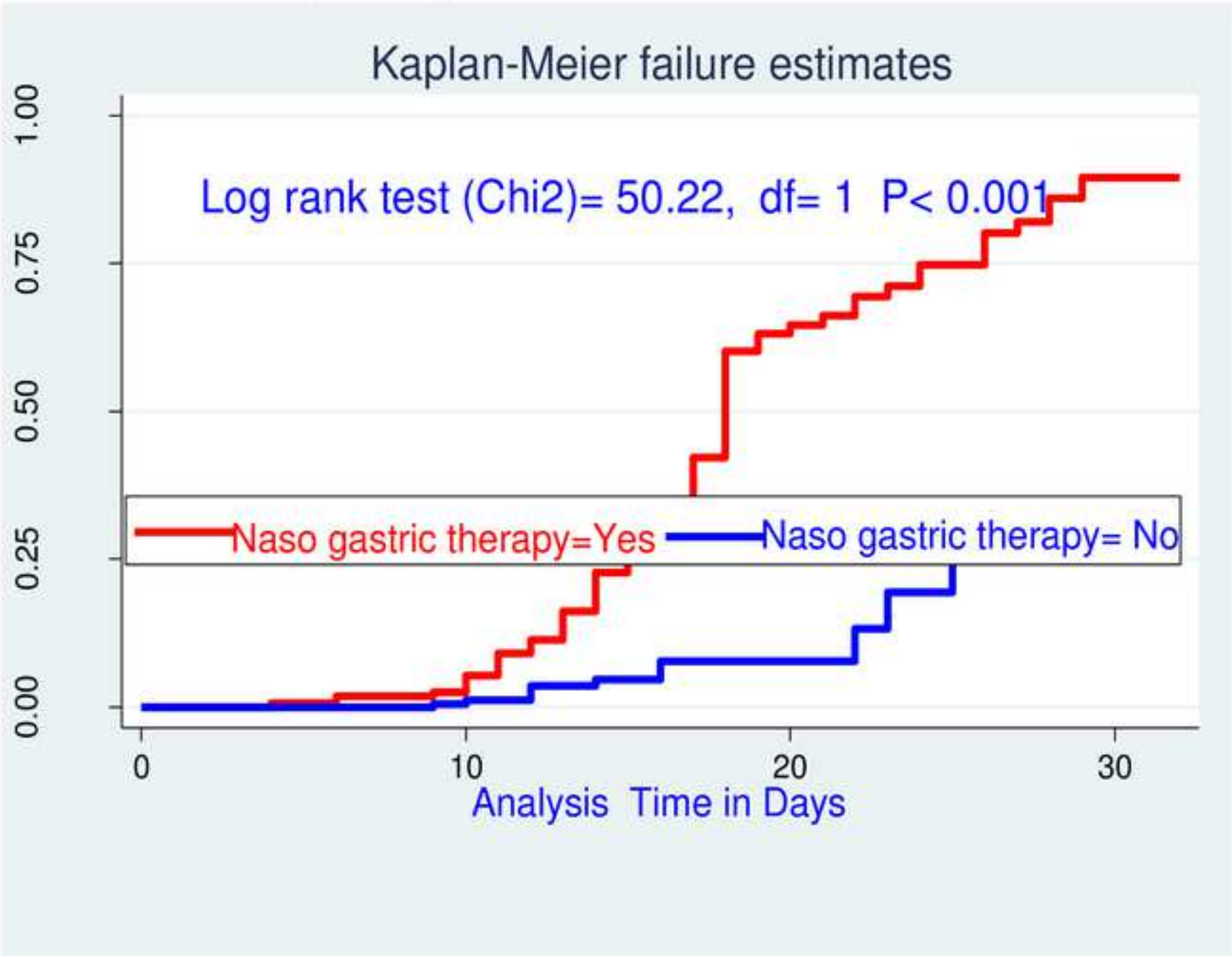

Click here to access/download;Figure;Figure 7.tif

**Fig**ure 7; Difference in Hazard of death based on hemoglobin status of SAM admitted children in Pawe general hospital since 2015-2019 (N=568).

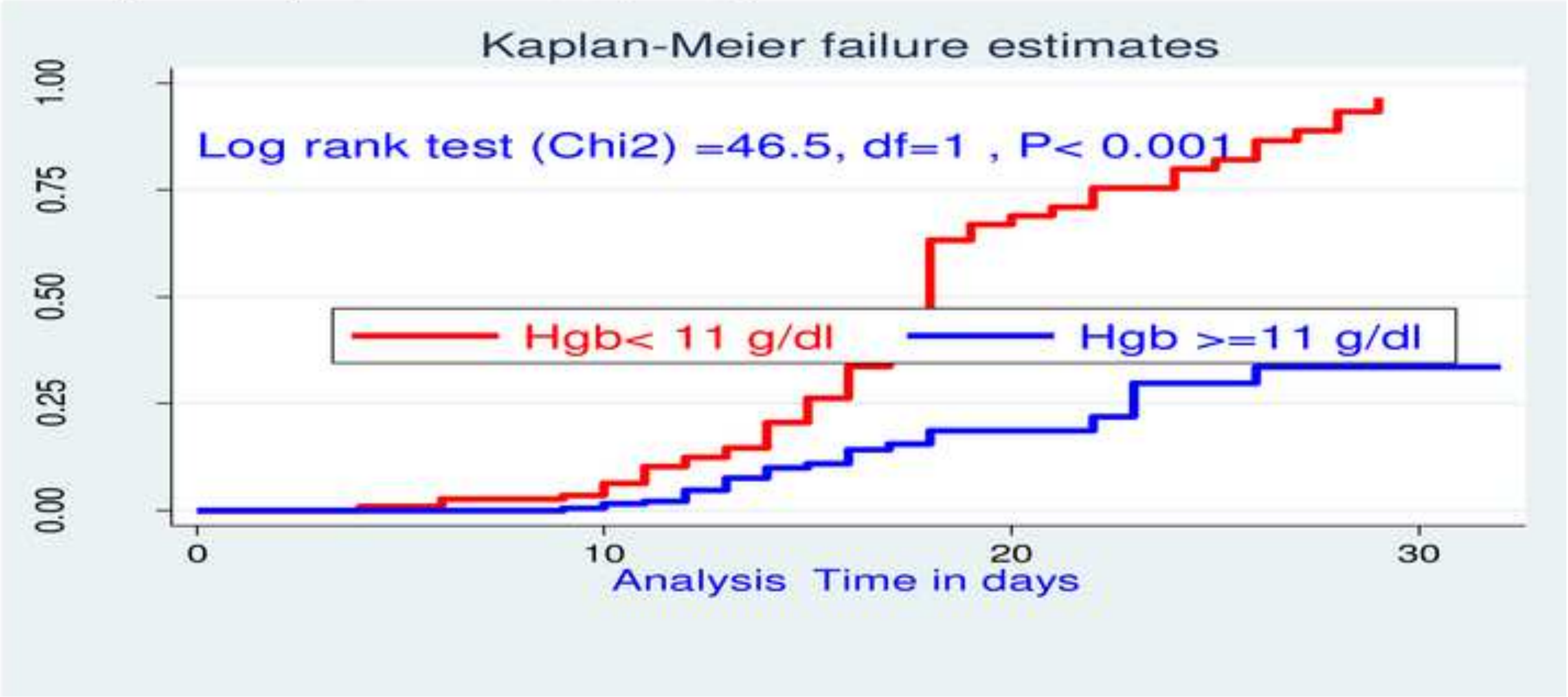

Click here to access/download;Figure;Figure 5.tif

**Figure5**; Difference in Hazard of death based on admission type children treated in Pawe general hospital since 2015-2019 (N=568).

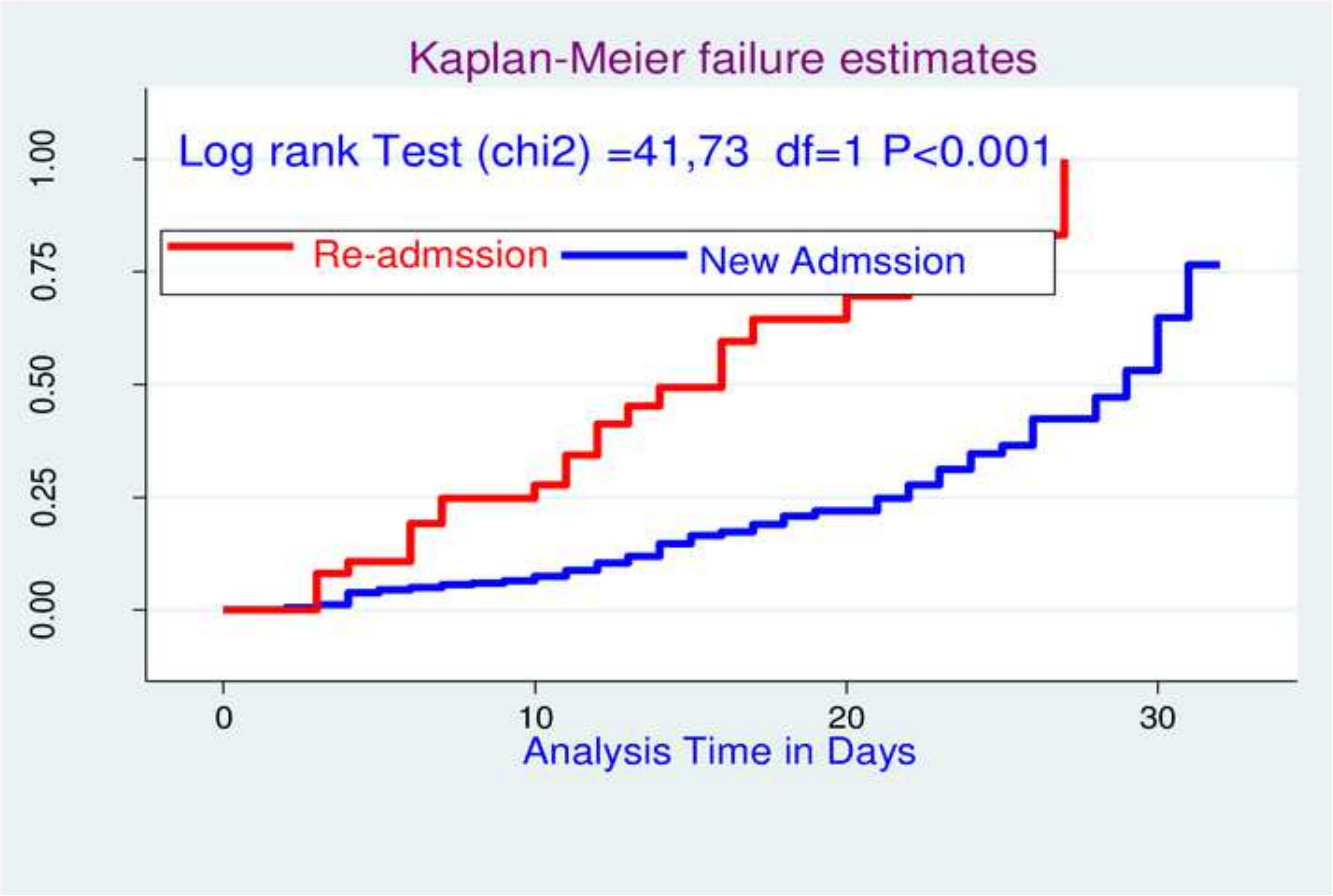

Click here to access/download;Figure;Figure 8.tif

**Fig**ure 8; Difference in Hazard of death based on diarrheao during SAM admitted children in Pawe general hospital since 2015-2019 (N=568).

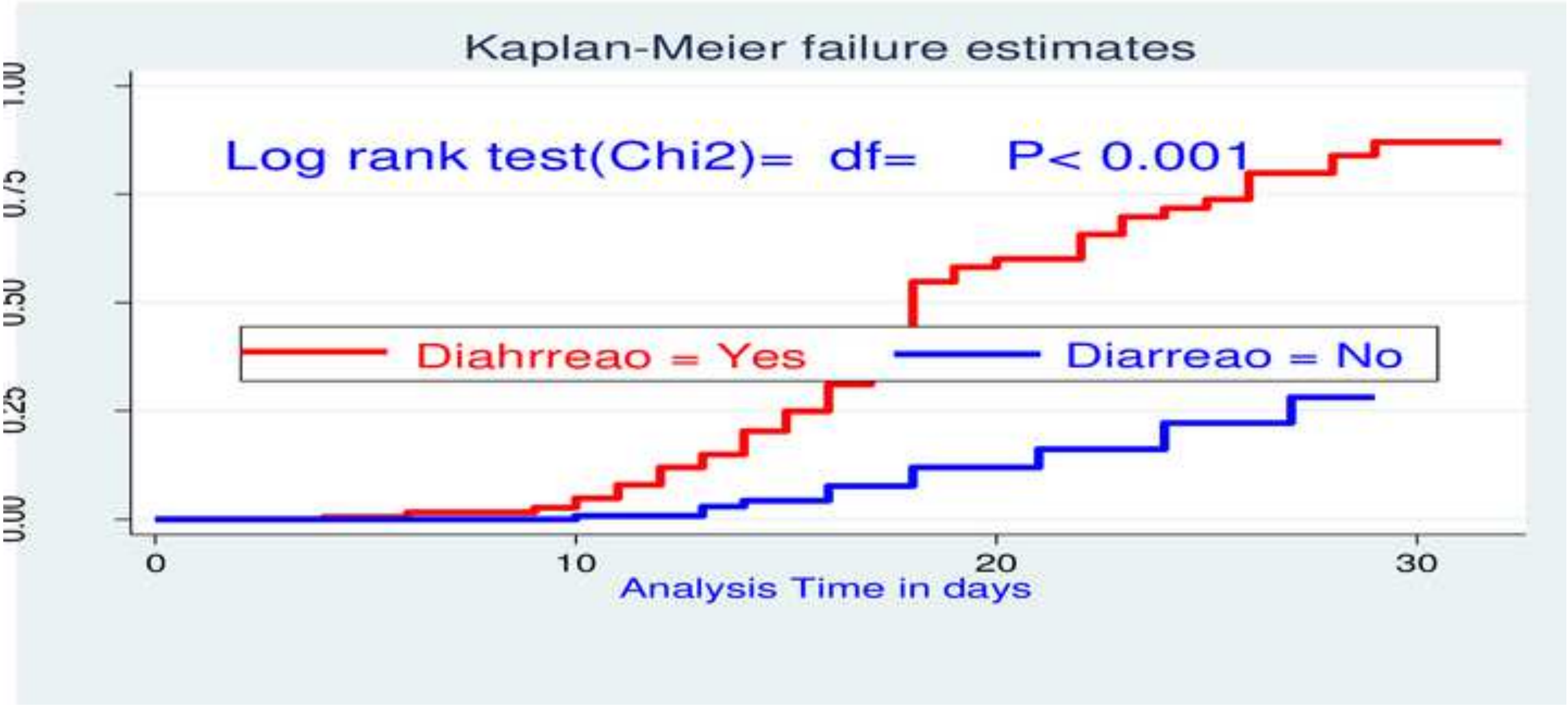

Click here to access/download;Figure;Figure 4.tif

Figure4; Difference in Hazard of death based on breast feeding status of SAM admitted children in Pawe general hospital since 2015-2019 (N=568).

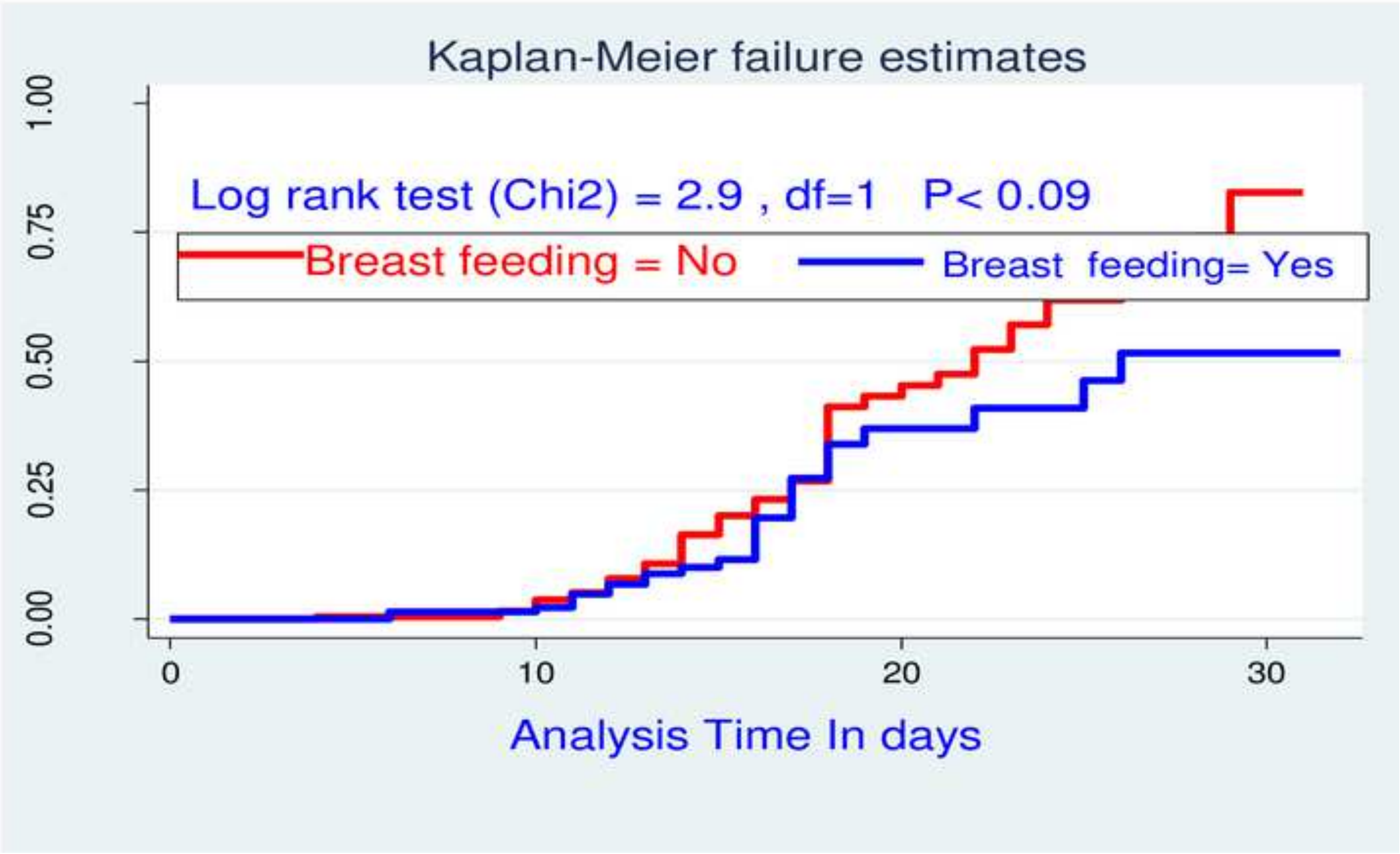

Click here to access/download;Figure;Figure 1.tif

**Figure.1 Number of severe acute malnutrition children addmited in Pawe General Hospital from 2015-2019**

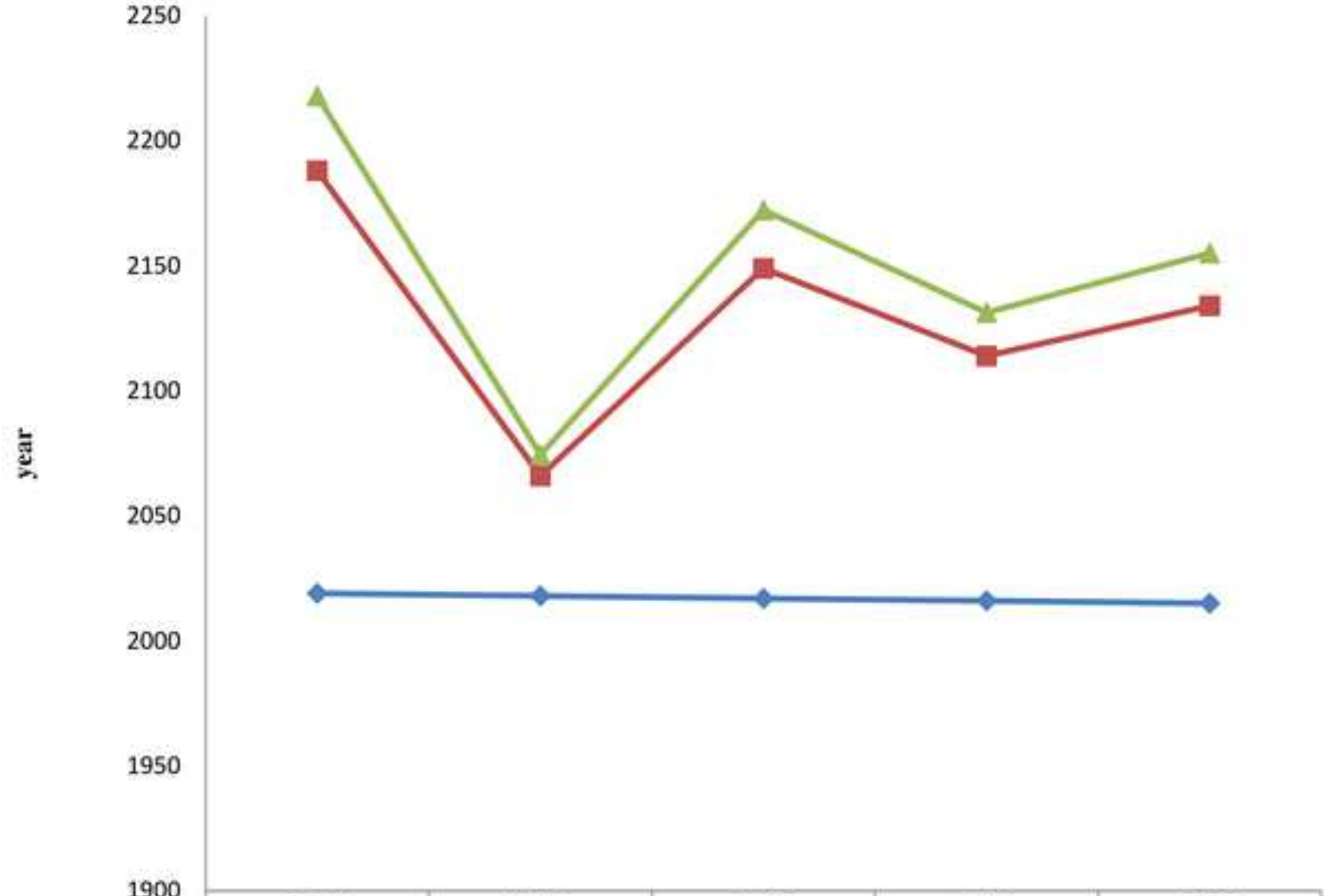


| | 2019 | 2018 | 2017 | 2016 | 2015 |
|---|---|---|---|---|---|
| percent | 29.86 | 8.48 | 23.32 | 17.31 | 21.02 |
| Frequency | 169 | 48 | 132 | 98 | 119 |
| YEAR | 2019 | 2018 | 2017 | 2016 | 2015 |

Click here to access/download
**Supporting Information**
English versions checklists.docx

English version of Data collection check lists

Click here to access/download

**Supporting Information**

English versions data collection checklists .docx

Final Data set(S1) as supporting information

Click here to access/download
**Supporting Information**
FINAL DATA SET OF SAM (S1).dta

# INCIDENCE AND PREDICTORS OF SEVERE ACUTE MALNUTRITION MORTALITY RATE AMONG CHILDREN AGED 6-59 MONTHS ADMITTED AT PAWE GENERAL HOSPITAL, NORTHWEST ETHIOPIA 2021

**Fassikaw Kebede (BSC, MPH) [1*] ,Belete Negese(BSc,Msc)[2], Tsehay Kebed(BSC,MA)[3] , Atitegeb Abera (BSC,MPH)[1], Getahun Fentaw(BSC,MPH)[1]**

1Department of Epidemiology & Biostatistics, School of Public Health, College of Health Science, Woldia University, Ethiopia 2021

[2]Dschools of Nursing and Midwifery, College of Health Science, Debre Birhan University, Ethiopia 2021

[3]Department of Geography & environmental study, College of social science, Bahirdare University Ethiopia 2021

**Authors email**

**FK: fassikaw123@gmail.com**
**BN: yabebalij@gmail.com**
**TK: tsehaynolawit@gmail.com**
**AA: atitegebabera@gmail.com**
**GF: gechfentaw1014@gmail.com**

**1*Corresponding Author**
**Fassikaw Kebede (BSC, MPH)**
**Lecturer at school of Public health**
**College of Health science Woldia University**
**FK = fassikaw123@gmail.com**
**Institutional email = fassikaw.k@wldu.edu.et**
**Web address URL: http://www.wldu.edu.et**

**Abstract:** Severe acute malnutrition (SAM) is defined by very low weight for height (below -3z scores of the median WHO growth standards), by visible severe wasting, or by the presence of nutritional edema. Mortality magnitudes for under-five children were well documented in Ethiopia, nevertheless lacking information on the predictors when death occurs during inpatient treatment. The aim of understanding to time when under-five children with inpatient SAM die and its predictors could be vital to develop the time-relevant intervention.

**Methods**: A retrospective cohort study was conducted among 568 under-five children from 1 January 2015 up to December 31, 2019, at Pawe general hospital. Epi Data version 3.2 and Stata/14 software were used for data entry and analysis, respectively. The Kaplan–Meier survival curves and log-rank test were performed to compare significant survival and death experiences among study participants. Bi-variables Cox-regression model was built to select candidate variables at *P*<0.25. Adjusted hazard ratio (AHR) with a 95% confidence interval (CI) was used to estimate a significant predictor for inpatient mortality at P<0.05

**Result:** - Five-hundred sixty-eight (N=568) admitted SAM cases were included in this study. The mean age of ~~the~~ participants was 27.4 (SD± 16.5) months the median time to death was 3 ~~with~~reported as 13 (IQR=8) days. The ~~-~~overall incidence density rate (IDR~~)~~) of ~~-of SAM~~ inpatient mortality was found ~~was~~16.03 per 100 (95%CI: 13.86; 20.04) person-days risk observation. Cases at baseline admitted with ~~Admission with~~ nasogastric therapy (NGT) (AHR=3.22; 95%CI; 1.65- 6.26, P<0.001), vomiting (AHR=5.1; 95%CI: 1.35-21.1, P<0.026), diarrhea (AHR=2.79; 95%CI: 1.46- 5.4; P<0.002), anemia (AHR= 1.89; 95% CI: 1.15- 3.2; P<0.012) , and re-admitted/relapsed SAM cases (AHR=1.7; 95%CI: 1.12 - 2.8, P<0.037) were significantly associated with inpatient mortality of SAM children.

**Conclusion:-**The overall SAM mortality rate was high among children treated in stabilizing center as compared with national standard of protocol(<11%). Cases at baseline admitted with vomiting, diarrhea, NGT, and re-admitted had highly associated for mortality incidence. Health education on early medical seeking behavior for care ~~and~~givers and adherence on the routine regimens may improve child inpatient treatment survival beside to boost up strategy to minimize attrition ~~after~~rate after case admission.

**Keywords**: Severe acute malnutrition, Predictors of mortality rate, Children, Ethiopia

## Introduction

Malnutrition remains one of the most common causes of morbidity and mortality in children throughout the world. It has been responsible for directly or indirectly 60%of the 10.9 million death annually among under-five children. Two-third of these deaths occurred during the first year of life **[1-4].** Childhood under-nutrition incorporates a combination of nutrition disorders that inclu~~ed~~ underweight, wasting, stunting, and micronutrient deficiency[5, 6]. Underweight (Weight-For-Age ≤- 3 Z score), is a composite measure of wasting and stunting, while wasting (Weight For Height ≤- 3 Z score) is acute malnutrition due to a recent failure to receive[illegible] nutrition's and may be affected by recent episodes of diarrhea and other acute illnesses[3, 5].

Although SAM usually affects all segments of a population, infants and young children are most vulnerable as they have higher nutritional requirements for growth and development[7]. . It is one of the leading causes of morbidity and mortality among infants and young children all over the world and more frequently in sub-Saharan Africa and south Asia[8]. The peak age for SAM is 6–18 months, which is the time of fast growth and brain development. However, it is increasingly becoming common that SAM may occur in infants less than six months of age

with many disadvantaged populations while starting to feed semi-solid and solid foods to children as young as two months[9]. Globally in 2018, 1 in 12 of the estimated 52 million children under five had SAM." ~~Globally in 2018, 52 Million under five years children in one in twelve of this age group are suffering from SAM~~ [10], and 2.9 million of those children were admitted for inpatient treatment ~~of SAM~~ [10, 11]. The Sub-Saharan African countries account for the most pro[illegible]ions of case fatality rate[12]. Despite the availability of outpatient treatment, the number of children with SAM seeking admission at stabilizing centers (SC) i[illegible]asing. However, 50% of children with SAM die due to inappropriate care during inpatient treatment[13]. One important reason is poor adherence for SAM therapeutic guidelines (a faulty case manag[illegible]ent) ~~The one underline reason is health care provider's poor adherence to world health organization SAM therapeutic guidelines~~ [1], and another is late presentation for treatment co[illegible]utes for high mortality burden ~~besides to late coming of the patient for treatment~~[14]. Malnutrition in Ethiopia is a long-term silent killing crisis, especially for infants, ~~and~~and children ~~children yet~~ contribut[illegible] an estimated 270,000 deaths ~~of under five children~~ in each year [15]. According to Health and Health-Related Indicators (HHRI) 2014, SAM was the third leading cause of mortality and accounted for 8.1%of the death of under-five children [5, 16, 17]. Despite Ethiopia launching the Seqota declaration to end death [illegible]AM ~~up severe acute malnutrition~~ ~~death by~~by 100% access to adequate food[18], SAM is still a reason for 20% of pediatric hospital admissions[19]. This is the main challenge of successful treatment outcome in developing countries like Ethiopia and generating a [illegible] based data for reason to death is highly important to meet the goal of therapeutic feeding centers. The aim of u[illegible]standing when inpatients death occurred during care and determining its predictors should be vital to develop the time-relevant intervention. So[illegible]s research was intended to come up with information about

Formatted: Font: (Default) Times New Roman

incidence of SAM mortality rate, and predictors for the time to death among children with severe acute malnutrition admitted to therapeutic feeding unit in Pawe general hospital.

## Methods

### Study setting, population, and design

A facility-based retrospective cohort study was conducted on [illegible]68 under-five children who were admitted for SAM treatment at Pawe General Hospital Metekel Zone Pawe woreda Northwest from 1st January 2015 up to December 31, 2019. Pawe general hospital is located 565 km away from Addis Ababa, the capital city of Ethiopia[20, 21]. [illegible]cording to the 2019 national population projection, this region has an estimated 1.21 million population[22]. The Pediatric ward is among the five inpatient departments found in Pawe general hospital with 252 beds for inpatient treatment ~~The Pediatric ward is among the five inpatient departments found in Pawe general hospital with 252 beds for inpatient treatment~~ [23, 24]~~.~~ and a separate stabilizing center for children with SAM.”~~Thought, the stabilizing center is separately built with single-based beds for children in SAM patient's treatments.~~ Ethiopia adopted a [illegible] of world health organizations for severe malnutrition treatment guidelines of under-five children using three phases; the first phase (phase I), the transition phase, and phase II. In all phases admitted children are treated empirically for infections, hypoglycemia and hypothermia and resuscitated to restore electrolyte balance.”~~In all phases admitted children were, treated and prevented for infections, resuscitated, restored electrolyte balance, hypoglycemia, and hypothermia~~. The [illegible]pital provides service for total catchment populations and nearby regions like Amhara both northeast and Westside. ~~It started providing ART service with aid from the Regional Health Bureau and the Ministry of Health in April 2007.~~ Following the time of SAM

admission, from 1st January 2015, up to 31st December 2019, there were a total of 578 Under-five children had been registered for SAM inpatient treatments.

**Sample size determination**

We determined the sample size for this study both by single population proportion and survival analysis formula using the following assumption; n =(Za/2)2P (1-P)/d2 and considering a 95% confidence level (Z a/2 = 1.96), the margin of error 5%, overall mortality rate 46%[6], and 15 % addition for incomplete data and yields 454. Whereas sample size for the second objective was achieved using STATA/SE version-14 by considering

Two-sided significance level (α = 5%),

$Za_{1/2}$ = Z value at 95% confidence interval = 1.96,

Power **($Z_B$)**=80%, and P = % cumulative occurrence of death rate, 1.65 HR.

The final sample siz (n) =$\frac{Event}{P(Event)} = \frac{(Z_{a/2}+Z_B)2}{\theta^2 p(1-p)} = \frac{(za/2+ZB)}{p(1-p)(lnHR)2}$ [25]

$\theta$ = ln(HR)

HR= $e^{\theta}$

e = event

P (e) = probability of event

P = Mortality rate *46%*from [6]

HR= hazard ratio (AHR)0.6 from [6].

By inserting all parameters into the STATA (SE)\14 gives 448. However, the five years multi-chart recoded reviewed only 578 records were there and those are manageable and included all for final analysis.

**Outcome ascertainment**

~~In this study,~~

"In this study, the outcome of interest was inpatient death due to SAM."

~~the outcome of interest is the SAM inpatient death~~, and death was defined as following after ~~inpatient~~ admission to report of death during treatment observation. Variables like age, sex, residence, medical comorbidity, vaccination status, vomiting, breastfeeding, NGT, medication

administration, admission types, malnutrition types, and mid-upper are circumference status, et[illegible] were considered as independent variables for the development of the checklist.

**Operational words**

**Admission criteria:-**According to WHO management of SAM a children who are 6–59 months of age, Weight-for-height ≤–3 Z-score, or mid-upper-arm circumference <115 mm, or presence of bilateral edema, and failed appetite test should be admitted for inpatient care[26].

**Discharged /declared Cured:** According to WHO management of SAM a children who are 6–59 months of age, during inpatient treatment declared as cured when weight-for-height/length is ≥–2 Z-score and they have had no edema for at least 2 weeks, or. The mid-upper-arm circumference is >115 mm and they may have had no edema for at least 2 weeks or [7].

**Defaulted/Abscond/Lost from following up:-**when severe acute malnutrition diagnosed &admitted children absent from stabilizing center for consecutive 2 days after started treatment[5].

**Anemia in children:-** was defined a[illegible]assified based on the mean amount of red blood cell or Hgb levels mainly classified into two ways: ~~No~~ anemia <11 and no anemia >=11 g/dL~~≥11 g/dL, and Anemia≤10.9 g/dl~~ [27].

**Data collection instrument and quality controls**

A structured English version checklist was developed and used for data extraction from admitted children's medical records on the Federal Ministry of Health (FMOH) severe acute malnutrition [7]. One day of training was given for three diploma nurses and One BSC public health officer for data collection tools, data extraction system, and the objectives of the study. To assure the quality of data, the data collection checklist was 5% of the total sample size was pretested. After the pretest, necessary modification of the data collection tool was incorporated. Strict follow-up

and supervision were carried out during data collection by principal investigators and feedback was given daily. The collected data were reviewed &checked for completeness before data entry.

**Data processing and analysis**

The collected data were entered using Ep-iData version 4.2 statistical software and exported to STATA (SE) R-14 version statistical software for further analysis. The proportional hazard assumption was checked for each variable and no variable was found with Schoenfeld residual test <0.05. The Kaplan–Meier survival curves and log-rank test were performed to compare significant experience among survival /death study participants.Variables with P-value < 0.25 in bi-variable Cox regression analysis were included into multivariable Cox regression model. Finally, a variable with an adjusted hazard ratio (AHR) it 95% confidence interval (CI) at P-value <0.05 were considered as significant predictors for inpatient mortality admitted children. The model fitness was checked using the Nelson- Aalen cumulative hazard rate relative to Cox-Snell residuals

**Ethical statement and consent to participation**

Ethical clearance was obtained from the institute of ethical review committee from Debre Markos University, College of Health Sciences (Ref. No: HSC/984/16/12). A formal letter was submitted to Pawe General Hospital for permission to be done entitled of the research articles, incidence, and predictors of severe acute malnutrition mortality rate children 6-59 months admitted in stabilizing center at Pawe general hospital, North West (2015-2019**).** Finally**,** Pawe hospital rechecked for ethical compatibility and permitted data access. According to national research ethical review guideline:-https://www.ccghr.ca/wp-content/uploads/2013/11/national-research-ethics-review-guidline[28] if the study was conducted through individual file records reviewed, no need for consent from the mothers or caregivers of the study subject. However, all

data s[illegible]ere kept confidential anonymously. The institution of the ethical review committee of Debre Marko's University was waived consent from the caregiver for all secondary data.

# Result:

**Baseline Socio-demographic and clinical characteristics**

After excluding 10(1.74%) individual files due to incompleteness, we reviewed 568 files of SAM cases admitted children registered for treatment from 1st January 2015 to December 31 /2020. Out of the included participants, slightly more than half, 324(57.04%) of partic[illegible] children were females, and m[illegible]ity, 356 (62.68 %) of them were f[illegible] in age group 6-24 months years. The mean age of the participants was children found 26.28 (SD = ±16.04) months. Seventy-eight percent of cases children were from r[illegible] resident, and majority of them 361(63.56%) of them them were on breast feeding during admission. Moreover, 169(29.86%) &119(21.02%) SAM cases were admitted during 2019&2015 years respectively, respectively **(Figure 1)**.

**De[illegible]ptive sever acute Malnutrition profiles**

Nearly four in five Majority, 457(80.46%) of t[illegible] participant cases were new sever acute malnu8tration cases admissions whereas; the remaining 111(19.6%) were relapsed / re-admitted SAM cases. Ofof the total 568SAM cases 318(55.9%) were admitted due to wasting; while the remaining 153(26.94), and 97(17.08) were due to Marasmus-Kwashiorkor and edematous (Kwashiorkor), respectively. [illegible]y seventeen percent (68.3% of wasting cases were observed in the 6-24 months age groups. Of the total of 97(17.08) edematous cases, nearly half (48.9%) of them were cured (**Table1).**

**Co-morbidity and Antibiotics**

The majority=,Moreover, [illegible]96(52.18%) of children had pneumonia during admission, while nearly two in five 217 (38.2%) of admitted cases had skin dermatitis. Of all 568 participants, 307 (54.35%) had multiple (more than two) comorbidity during admission, for instance, SAM with diarrhea (11.17%) plus pneumonia (10.55%), and SAM with anemia (19.3%) plus vomiting (14.8%). More than half of 53.32% of the admitted cases had altered body temperature (≥37.5 °C). In addition, 470 (82.75%) and 242 (42.61%) children received vitamin A and deworming syrup during inpatient care, while only 22.8% of children received intravenous fluid **[illegible]le 2).**

**Treatment outcomes of the stabilizing centers**

At the end of this study period, 326 (57.39%) admitted under-five children had been cured, while the remaining 106 (18.66 %) lost from follow-up, 46 (8.10%) transferred out, and the remaining 91 (16.03%) children died. D[illegible]g in-patient treatment of cases,–majority of deaths, 38 (41.11%), were reported during Phase I within 24-144 hours after admission whilst 124 (38.6%) of SAM children were cured in phase II (Table3). "

the majority of 38(41.11%) deaths were reported at Phase I within 24-144 hours after admission. Inversely, 124(38.6%) SAM admitted children declared as cured at phase II **(Table3).**

**Incidence rate of SAM mortality**

The study participants were followed for 5108 Person per Month (PMOS) risk observation. The overall incidence of the mortality rate was 16.03 per 100 (95%CI: 13.86; 20.04) person-day observations**.** The median duration from admission to death was 13 (IQR=±8) days. The proportion of new death during inpatient treatment at the end of 1st week, 2nd week, 3rd week, and end of follow up were found, 28/91(30.76%),26/91(28.57%), 16/91(17.5%), 18/91(19.78%), and 3/91(3.3%), respectively.

**The log-rank estimate of mortality and hazard difference**

The log-rank test estimate revealed that the mortality among SAM admitted children were significantly varied among the covariates. The Kaplan Meier survival curve together with the log-rank test shows different in hazards of death for SAM admitted children on different covariate (**Table4).**

Baseline categorical variables like anemia, vomiting, diarrhea, and NGT during admission had significant survival differences when compared to their counterpart. *For instance, the mean survival time for those who had anemia during admission was 18.6 (95%CI; 17.38, 19.9) days and it was 27.5 95%CI; 25.8  29.1) days for who had no anemia during SAM admission (P-value, <0.0001) (Figure??)*

*Likewise, the mean survival time of those who had no diarrhea was 27.8 (95%CI; 26.04, 29.1) days while it was 12.2(95%CI; 9.0, 14.81) days for those who had diarrhea (p-value<0.001).The mean survival time for new admitted SAM children was 24.4(95%CI; 22.9, 25.7) days, while it was  6.4 (95%CI; 4.6, 8.2) days (p-value<0.0001)*.

**(Figure 2-5).**

**Predictors of SAM Mortality**

During bi-variable Cox regression analysis was running 17 variables were run and subsequently, 11 variables were transferred into multivariable Cox regression by p-value <0.25 criteria of regression. After adjustment of potential confounding, five variables found significant predictors for SAM inpatient mortality. The risks of death for children admitted with NGT was three times (AHR=3.22; 95%CI; 1.65- 6.26, P<0.001) increased as compared with children without NGT.

Death risk for Re-admitted SAM diagnosed cases were nearly two-times increased as compared with new admission case (AHR=1.7; 95%CI: 1.03—2.8, P<0.037). Hazards of death for children having vomiting during admission was five times increased as compared with no vomiting case at admission nearly five times (AHR=5.1; 95%CI:1.35—21.1, P<0.026). Likewise, the death hazard for children admitted with anemia was twice increased as compared with children with no anemia (AHR= 1.89; 95% CI: 1.15—3.2; P<0.012). The risk of death for SAM diagnosed and admitted children with diarrhea was nearly three times higher as compared with no diarrhea at admission (AHR=2.79;95%CI:1.46--5.4 ;P<0.002) **(Table 5).**

## 4.0. Discussion

This study was primarily i[illegible]ded to estimate incidence of inpatient SAM mortality rate and its potential predictors after admitted children in stabilizing center registered from 1st January 2015 up to December 31, 2019in pawe general hospital.the study have indicated that , at the end of follow up

~~At the end of the study~~ peri~~od,~~ 326 (57.39%) SAM admitted children had cured, while the remaining 106 (18.66 %) lost from follow-up, 46 (8.10%) transferred out, and 91(16.03%) cases were died. The overall incidence density rate (IDR) of ~~SAM~~ mortality was 16.03 per 100 (95%CI: 13.86; 20.04) person-days observations.

**Formatted:** Font: (Default) Times New Roman, 12 pt

**Formatted:** Font color: Black

**Formatted:** Justified, Space Before: 0 pt, After: 0 pt, Line spacing: Double, Pattern: Clear, Tab stops: 6.13", Left

-This was higher than the national SPHERE reference (<11%) [12] and might be due to delayed presentation to the health institution (SC), early discontinuation of treatment due to insufficient financial means admissio[illegible]5] , and poor adherence to WHO SAM guidelines [1, 29].

How[illegible], our finding is not consistent and higher than ~~with~~ finding reported in Mekele hospital 3.8%[3], Tigray general hospitals 6.65%[3], Dilla hospital 7.57%[1], Bahir-dare referral hospital 7.7%[30], Southern hospital 10.8%[31]. The difference might be due to al[illegible]se are specialized universities and referral hospitals, which provide excellent and comprehensive treatment care, unlike the counterpart of the pastoralist community our study general hospitals.

In[illegible]~~n,~~ sum, the median time to death was reported as 13 (IQR=8) days. This is ~~was~~ not consistent and highe~~r~~ than reported in Gondere ~~specialized university~~ hospital 12 days[5], but lower than findings in southern hospitals 17 days [31], Mekele referral hospital 41.2 days[3], and Shebedino hospital 36 days [2]. Unfortunately, the economic constraints of ca[illegible]upporters to buy additional drugs and food exposed for early loss from inpatient care. This is a real scenario in our study setup; more than 18.66%of admitted cases were lost from follow-up.

~~Regarding~~Regarding ~~predictors~~to predictors for inpatient ~~of~~ mortality, the hazard of death for anemic children during admi[illegible]n was two-fold increased as compared wi[illegible]e counter group. This is comparableand consistence with the finding in Gonder referral hospitals [5], and Nekemte Referral Hospital ~~(NRH)~~ [32]. I[illegible]trast to ~~to~~our study reported ~~this~~, the study result have no association role for anemia prevention by oral medications( e.g., vitamin A= 82.75% and folic acid =76.94 %), but a study result in NRH [32] revealed a significant association dearth of deworming (P=0.031) prevention versus anemia incidence (0.042) [32].

Formatted: Font: (Default) Times New Roman, 12 pt

A child may experience more than one episode of SAM, depending on the improvement of the underlying factors during inpatient treatment . A child may experience more than one episode of SAM relapsing, depending on the improvement of the underlying factors during inpatient [33] in line to this -,our research finding revealed SAM relapses have a two-fold hazard of death.

In line with the study - conducted at Northwest Ethiopia –finding in Lusaka, Zambia [34], Gonder referral hospitals [35], Southern Ethiopia. [2], and Irena [34],SAM children admitted with baseline diarrhea had an increased risk of death, consistent with our two-fold increase in death, which may be due to combination of fluid and electrolyte loss and possible hypovolemic shock. Moreover, the hazard of death among children with vomiting at admission is three times increased as compared with no vomiting case.This is Consistent with the study finding at North Gonder [35]. Both vomiting and diarrhea are associated with shifting of physiological fluids homeostatic and easily loss of continuity for children whatever medication administration orally. This could be due to the shrinking of the intracellular potassium pumping balance of the body homeostatic. On the other hand, the hazards of death for children at baseline admitted with nasal-gastric therapy (NGT) were two times increased as compared with no NGT admissions. UnlikeUnlike in our researches research findings hospitals of Fikre et. al[30], there is no positive association between admitted SAM cases with shock, vomiting, and poor adherence, however study finding in Dilla referral hospitals [13], and Gonder referral Hospitals [5] had significant associations of inpatient mortality with Vomiting (P<0.001), altered breathing(P<0.001), and admission with NGT (P<0.038).

**Formatted:** Font: (Default) Times New Roman, 12 pt

**Formatted:** Font: (Default) Times New Roman, 12 pt

**Limitations**

The possible limitation of this study is that since it is a record review, it failed to consider a broad range of factors like some biochemical indices and the socioeconomic status of care givers. Therefore, the interpretation and application of the finding for decision and policy direction should account for these inherent limitations of the study.

~~it failed to consider a broad range of factors like some biochemical and individual bases economic status of caregivers' might introduce a high array of missing confounders. As such, the interpretation and application of the finding for decision and policy direction should account for these inherent limitations of the study~~.

**Conclusion:** The overall SAM mortality rate was high among children treated in stabilizing center as compared with national standard of protocol. Cases at baseline admitted with vomiting, diarrhea, NGT, and re-admitted had highly associated for mortality incidence. Health education on early medical seeking behavior and adherence on the routine regimens may improve child survival beside to boost up strategy to minimize attrition after admission

**Abbreviation**

AHR, adjusted hazard ratio; CHR, crude hazard ratio; CI, confidence interval; FMOH, Ethiopian Federal Ministry of Health; MUAC, mid-upper arm circumference; SC, stabilizing center; SAM; severe acute malnutrition; NGT, nasal gastric intubation for feeding; WFH, weight for height; SD, standard deviation.

**Acknowledgment**

The authors would like to acknowledge Pawe General Hospital's administrative staff.

**Supporting information**

All relevant data are within the paper and its Supporting Information files.

**Formatted:** Font: (Default) Times New Roman, 12 pt

With reasonable request, we will send it is in the hand of the main authors

**Conflict of interest**

The authors declare that there are no conflicts of interest for this study

**Funding**

There is no especial grant of funding for this research from any organization

**Author's contribution**

Conceptualization; Fassikaw Kebede Tsehay kebede

Data curationcu ration: Fassikaw Kebede Tsehay kebede

Formal analysis; Fassikaw Kebede, Tsehay kebede

Methodology; Fassikaw Kebede,

Software ;Software; Fassikaw Kebede, Belete Negese, Tsehay kebede

Supervision; Fassikaw Kebede Getahun Fentaw

Writing – original draft: Fassikaw Kebede, Getahun Fentaw

Writing – review & editing: Fassikaw Kebede,Belete Negese, Tsehay kebede, Atitegeb Abera ,

## Reference

1. Tadele Girum, M.K., BEFIKADU TARIKU, *Incidence and Predictors of Mortality among Severe Acute Malnourished Under Five Children Admitted to Dilla University Referal Hospital: A*

*Retrospective Longitudinal Study.* Journal of Biology, Agriculture and Healthcare, 2016. **Vol.6, No.13, 2016**(ISSN 2224-3208 (Paper)).
2. Genene Teshome1, T.B.a.S.G., *Time-to-recovery from severe acute malnutrition in children 6–59 months of age enrolled in the outpatient treatment program in Shebedino, Southern Ethiopia: a prospective cohort study.* BMC Pediatrics, 2019. **13**(https://doi.org/10.1186/s12887-019-1407-9).

3. Gebremicael Guesh1, G.D., Mebrahtu Abay3, Berhe Beyene3, Ermyas Brhane3* and Kalayu Brhane4, *Survival status and predictors of mortality among children with severe acute malnutrition admitted to general hospitals of Tigray, North*

*Ethiopia: a retrospective cohort study.* BMC Research Notes, 2018. **https://doi.org/10.1186/s13104-018-3937-x**.
4. (WHO), w.h.o., *Key malnutration gelobal report* Malnutration report, 2018.
5. Fasil Wagnew, D.T., Mengistu Mekonnen and Amanuel Alemu Abajobir, *Predictors of mortality among under-five children with severe acute malnutrition, Northwest Ethiopia: an institution based retrospective cohort study.* Archives of Public Health 2018. **(2018)** (https://doi.org/10.1186/s13690-018-0309-x).
6. Tendai Munthali, C.J., Lungowe Sitali, Rosalia Dambe, and Charles Michelo, *Mortality and morbidity patterns in under-five children with severe acute malnutrition (SAM) in Zambia: a five-year retrospective review of hospital-based records (2009–2013).* ARCHIVES OF PUBLIC HEALTH (2015), 2015. **DOI 10.1186/s13690-015-0072-1**.
7. FMOH, *PROTOCOL FOR THE MANAGEMENT OF SEVERE ACUTE MALNUTRITION CHILDREN IN ETHIOPIAN CONTEXT.* SAM managment manual, 2007.
8. Ltd, D.I.P.R., *Global_Nutrition_Report SHINING A LIGHT TO SPUR ACTION ON NUTRITION.* Report Acesed10 septemebr 2021, 2018.
9. Wagnew Tesfay1, M.A., Solomon HintsaID2, Tekia ZafuID2*, *Length of stay to recover from severe acute malnutrition and associated factors among under-five years children admitted to public hospitals in Aksum, Ethiopia.* PLOS ONE, 2020. **e0238311.https://doi.org/10.1371/journal.pone.0238311**.
10. UNICEFandWorldhealthorganizarion, *KEY FINDING &LEVELS AND TRENDS IN CHILD MALNUTRITION.* 2018
11. DOCUMENT, U.P.G., *MANAGEMENT OF SEVERE ACUTE MALNUTRITION IN CHILDREN: WORKING TOWARDS RESULTS AT SCALE.* Mnaule 2014.
12. AustralianAgencyforInternationalDevelopment, *SPHEER project in humanitorian charter and minimum standred in humanitorian responsen.* Guideline, 2011. **Third edition** (ISBN 978-1-908176-00-4): p. 402.
13. Girum, T., et al., *Survival status and predictors of mortality among severely acute malnourished children <5 years of age admitted to stabilization centers in Gedeo Zone: a retrospective cohort study.* Ther Clin Risk Manag, 2017. **13**: p. 101-110.
14. Banga, D., et al., *Comorbidities and Factors Associated with Mortality among Children under Five Years Admitted with Severe Acute Malnutrition in the Nutritional Unit of Jinja Regional Referral Hospital, Eastern Uganda.* Int J Pediatr, 2020. **2020**: p. 7809412.
15. Habtemu Jarso , A.W.a.F.A., *Survival status and predictors of mortality in severely malnourished children admitted to Jimma University Specialized Hospital from 2010 to 2012, Jimma, Ethiopia: a retrospective longitudinal study.* BMC Pediatrics 2015. **15**(DOI 10.1186/s12887-015-0398-4).
16. ETHIOPIA, F.D.R.O., *Seqota Declaration Innovation Phase Investment Plan 2018 - 2020.* MANUAL, 2018.
17. Nigatu†2, M.a.T.H., *Modeling trends of health and health related indicators in Ethiopia (1995-2008): a time-series study.* Health Research Policy and Systems, Accepted: 13 December 2009. **7:29 doi:10.1186/1478-4505-7-29**.
18. CoHA, *Social and Economic Impact of Child Undernutriti in Egypt, Ethiopia, Swaziland*

*and Uganda.* Report, 2010.

19. Fasil Wagnew1*, D.T., Mengistu Mekonnen2 and Amanuel Alemu Abajobir3, *Predictors of mortality among under-five children with severe acute malnutrition, Northwest Ethiopia: an institution based*

*retrospective cohort study.* Archives of Public Health (2018) 76:64, 2018. **https://doi.org/10.1186/s13690-018-0309-x**.

20. Fassikaw Kebede1, Nemera Eticha2, Belete Negese3, Mastewal Giza1, Tadesse Tolossa4 and Bizuneh Wakuma5, *Predictors for a Cure Rate of Severe Acute Malnutrition 6-59 Month Children in Stabilizing Center at Pawe General Hospital, Northwest Ethiopia: Retrospective Cohort Study.* International Journal of Child Health and Nutrition, 2021, 10, 34-43, 2021. **10**(https://doi.org/10.6000/1929-4247.2021.10.01.5): p. 10.

21. Fassikaw Kebede , B.K., 2 Tsehay Kebede ,3 and Melaku Agmasu4, *Effect of Isoniazid Preventive Therapy on the Incidence of Tuberculosis among Seropositive Children Attending HIV/AIDS*

*Care in Two General Hospitals, Northwest Ethiopia, 2021.* Hindawi

Journal of Tropical Medicine

Volume 2021, Article ID 9996953, 9 pages

https://doi.org/10.1155/2021/9996953, 2021.

22. UNICEF, *Situation Analysis of Children and Women:Benishangul-Gumuz Region.* REPORT 2019.

23. Fassikaw Kebede, D.J.a.A.A., *Time to Develop Pulmonary Tuberculosis and Predictors among HIV Infected Children Receiving Anti-Retroviral*

*Therapy in Assosa and Pawe General Hospitals, North West Ethiopia: A Retrospective Cohort Study.* Journal of Pulmonary & Respiratory Medicine, 2020. **10**(ISSN: 2161-105X): p. 10.

24. Csaba Magyar a, M.M.G.b., Zolan Savoly a, Istvan Simon *The role of stabilization centers in protein thermal stability.* Biochemical and Biophysical Research Communications, 2016. **471 (2016) 57e62**(http://dx.doi.org/10.1016/j.bbrc.2016.01.181).

25. Moore, D.F., *Dirk F. Moore: Applied Survival Analysis Using R. Piscataway.* , NJ, USA: Springer Nature; 2015., 2015.

26. worldHealthorganization(WHO), *Updates on the management of severe acute malnutrition in infants and children.*

*Geneva: World Health Organization; 2013.* Guideline:, 2013.

27. Deepak Dwivedi, V.S.J.S. and Sangita Sharma, *Study of Anaemia in Children with Severe Acute Malnutrition.* J. Nepal Paediatr. Soc., 2017. **/Vol 37/Issue 3**(doi: http://dx.doi.org/10.3126/jnps.v37i3.18480).

28. EthiopianMinistry of Science and Technology, *Ethiopian National-research-ethics-review-guidline.* Book, published 2014, acccesed 2021.

29. Tadele Girum1, E.M.A.W., *Comparative Analysis of the Survival Status and Treatment Outcome of Under-five Children Admitted with Severe Acute Malnutrition Among Hospital-based and Health Center Based Stabilization Centers, South Ethiopia.* The Open Public Health Journal, 2018. **11**(DOI: 10.2174/1874944501811010209): p. 10.

30. Hanna Demelash Desyibelew1, A.F., Haile Woldie3, *Recovery rate and associated factors ofchildren age 6 to 59 months admitted with severe acute malnutrition at inpatient unit ofBahir Dar Felege Hiwot Referral hospital*

*therapeutic feeding unite, northwest Ethiopia.* PLOSONE, 2017. **| DOI:10.1371/journal.pone.0171020 F**.

31. Fikrie, A., A. Alemayehu, and S. Gebremedhin, *Treatment outcomes and factors affecting time-to-recovery from severe acute malnutrition in 6–59 months old children admitted to a stabilization center in Southern Ethiopia: A retrospective cohort study.* Italian Journal of Pediatrics, 2019. **45**(1).
32. Muluken Berhanu Mena, M.G.D., 2 and Bruke Berhanu Billoro3, *Treatment Outcome of Severe Acute Malnutrition and Its Determinants among Pediatric Patients in West Ethiopia.* Hindawi Publishin International Journal of Pediatrics, 2018, (Article ID 8686501, 7 pages).
33. Abera LambeboID1*, D.T., Tefera Belachew2, *Frequency of relapse for severe acutemalnutrition and associated factors among under five children admitted to health facilities in Hadiya Zone, South Ethiopia.* PLOS ONE 2021(https://doi.org/10.1371/journal.pone.0249232 M).
34. Abel H Irena1*, M.M.a.V.M., *Diarrhea is a Major killer of Children with Severe Acute Malnutrition Admitted to Inpatient Set-up in Lusaka, Zambia.* BMC Nutrition Journal 2011, 10:110, Irena et al. Nutrition Journal 2011, 10:110

**10:110**(http://www.nutritionj.com/content/10/1/110).
35. Mamo, W.N., et al., *Time to recovery and determinants of severe acute malnutrition among 6–59 months children treated at outpatient therapeutic programme in North Gondar zone, Northwest Ethiopia: a prospective follow up study.* Italian Journal of Pediatrics, 2019. **45**(1).

**Table1:** Socio-demographic and clinical characteristics of SAM admitted children in Pawe General Hospital, North West Ethiopia (2015-2019)(n=568).

| Variables | Characters | Frequency % |
|---|---|---|
| Sex | Male | 244(42.96) |
| | Female | 324 (57.2) |
| Residence | Urban | 126(22.18) |

| | | |
|---|---|---|
| | Rural | 442(77.82) |
| Age | Between 6-24 month | 355(62.68) |
| | Between 24-48 month | 160(28.17) |
| | Above ≥48 month | 53(9.15) |
| SAM types | Wasting(Marasmic) | 318(55.99) |
| | Marasmus Kwashiorkor | 153(26.94) |
| | Kwashiorkor( Edematous ) | 97(17.08) |
| Skin dermatitis | Absent | 353(61.8) |
| | Present | 217(38.2) |
| Vomiting | Absent | 281(47.89) |
| | Present | 296(52.13) |
| Altered body temperatures ≥37.5C$^0$ | Absent | 265(46.65) |
| | Present | 303(53.35) |
| Diarrheal | Absent<br>Present | 257(45.2)<br>311(54.75) |
| Pneumonia | Absent | 272(47.89) |
| | Present | 296(52.11) |
| Anemia | Absent | 399(70.75) |
| | Present | 169(29.75) |
| Breastfeeding status | No breastfeed | 207(36.4) |
| | Yes breastfeed | 361(63.56) |
| Admission type | New admission | 457(80.46) |
| | Re-admission | 111(19.54) |
| Nasogastric tube during admission | Present | 243(41.02) |
| | Absent | 325(58.98) |
| Vitamin A supplementation | Given | 470 (82.7) |
| | Not given | 98( 17.25) |
| Folic acid | Given | 406 ( 71.48 ) |
| | Not given | 162 (28.52 ) |
| Blood transfusion during admission | Given | 123 (21.65) |
| | Not given | 445 ( 78.35) |

**Table2**:-Performance indicator of stabilizing center with compared *Sphere reference* for Incidence rate and predictors of Under-five SAM children in Pawe General Hospital (n=568).

| **Performance indicator** | **Five years Performance of Stabilizing center** | **SPHERE project reference value** |
|---|---|---|

| | | Overall | Acceptable | Alarming |
|---|---|---|---|---|
| Treatment cure rate | 57.38% | 77.9% | >75% | <50% |
| Incidence of death rate(IDR) | 16.7% | 12.3% | <15% | >25% |
| lost from follow up | 18.36% | 5.5% | <10% | >15% |
| Transfer out | 8.7% | 4.8% | ____ | ____ |

Table3: Proportions of SAM treatment outcome by phase after being admitted to stabilizing center in Pawe general Hospital (2015-2019 (n=568).

| Indicators | **Phase 1** | **Transition phase** | **Phase 2** | **Treatment outcome by phases(N=568)** |
|---|---|---|---|---|
| **Death rate** | **38(41.11)** | **24(26.4)** | **29(31.50)** | **91** |
| **Treatment cure rate** | **97 (29.56)** | **105(32.39)** | **124(38.60)** | **326** |
| **Lost follow up** | **9(8.50)** | **34 (32.81)** | **63 (60.0)** | **106** |
| **transferred out** | **3(6.52)** | **5(10.8)** | **38(82.6)** | **46** |

Table-4: Log rank estimate of variables among SAM admitted children at Pawe General Hospitals in North West Ethiopia, 2021.

| ***Categories*** | **log rank test estimated** | **P - Value** |
|---|---|---|
| Age(years) | $X^2 = 64.36$ | **P-value = 0.053** |
| Sex(Male/ female) | $X^2 = 2.71$ | **P -value = 0.099** |
| Residence(Urban /rural ) | $X^2 = 3.23$ | **P -value = 0.63** |

| | | |
|---|---|---|
| Admission types(New/ Re-admission) | **$X^2$ = 37.02** | **P - value = 0.001** |
| Had NGT during admission (Yes/No) | **$X^2$ = 50.22** | **P -value = 0.001** |
| Measles vaccine (Taking /Missed) | **$X^2$ = 10.98** | **P -value = 0.009** |
| Iv fluid (Given/No given) | **$X^2$ = 2.38** | **P -value = 0.123** |
| Parenteral Antibiotics (Given/ Not given ) | **$X^2$ = 9.16** | **P -value = 0.003** |
| Vitamin A supplementation (Given/Not Given ) | ***$X^2$ =1.82*** | **P -value =1.7891** |
| Anemia during admission (Present /Absent ) | ***$X^2$ = 46.89*** | **P -value =0.001** |
| Folic acid supplementation (Given /Not Given) | ***$X^2$ =2.016*** | ***P -value =0.152*** |
| Vomiting during admission (Present /Absent ) | ***$X^2$ =23.02*** | ***P -value =0.001*** |
| Diarrhea during admission (Present/Absent) | ***$X^2$ =38.9*** | ***P -value =0.001*** |
| Comorbidity during admission (Present /Absent) | ***$X^2$ =2.15*** | ***P -value =0.645*** |

**Table 5: Bi-variable and multivariable Cox regression for predictors of SAM mortality among children to Pawe general hospital since 2015-2019 (n=568), North West Ethiopia.**

| Covariate | Categories | Survival status | | | | |
|---|---|---|---|---|---|---|
| | | Death | Censored | CHR(95%CI) | AHR(95%CI) | P-value |
| **Age of children** | 6-24 Month | **68** | **186** | **1** | | |
| | 24-48 month | **22** | **145** | **0.9(0.57--1.4)** | **1.2(0.73-- 2.01)** | **0.443** |
| | ≥48 month | **1** | **46** | **0.61(0.59 -4.4)** | **2.1 (0.82- -3.9)** | **0.27** |
| **Sex** | Male | **30** | **214** | **1.13(0.89--1.43)** | **0.96 (0.61 --1.53)** | **0.859** |
| | Female | **61** | **263** | **1** | **1** | |
| **Admission types** | **New** | **67** | **183** | **1** | **1** | |
| | **Re-admission** | **24** | **294** | **1.7(2.4 --- 6.1)** | **1.7 (1.03—2.8)** | **0.037*** |
| **NGT during Admission** | **Yes** | **79** | **164** | **2.3(2.3--6.99)** | **3.22(1.65—6.26)** | **0.001*** |
| | **No** | **12** | **313** | **1** | **1** | |

| | | | | | | |
|---|---|---|---|---|---|---|
| **Vomiting** | **Yes** | **88** | **208** | **2.34(1.28 ---4.3)** | **5.1(1.35---21.2)** | **0.026*** |
| | **No** | **3** | **269** | **1** | **1** | |
| **Anemia** | **Present** | **64** | **105** | **4.2(2.6---3.67)** | **1.89(1.15–3.12)** | **0.012*** |
| | **Absent** | **27** | **372** | **1** | **1** | |
| **Diarrhea** | **Yes** | **79** | **232** | **5.6(2.99—10.6)** | **2.79(1.46—5.4)** | **0.002*** |
| | **No** | **12** | **245** | **1** | **1** | |

**Version =4**
**Date = 17 September2021**

**Author's response to reviews**

**Titles:-INCIDENCE AND PREDICTORS OF SEVERE ACUTE MALNUTRITION MORTALITY RATE AMONG 6-59 MONTHS CHILDREN IN PAWE GENERAL HOSPITAL NORTHWEST ETHIOPIA 2021**

**Authors email**

1) **Fassikaw Kebede:-fassikaw123@gmail.com**
2) **Belete Negese: - yabebalij@gmail.com**
3) **Tsehay kebede: - tsehaynolawit@gmail.com**
4) **Atitegeb Abera:-atitegebabera@gmail.com**
5) **Getahun Fentaw:-gechfentaw1014@gmail.com**

*DR Walter RJ Taylor Comment*
*Academic editor of* ***PLoS ONE Journal***

## Additional Editor Comments PONE-D-21-11027-R3 Submission

*INCIDENCE AND PREDICTORS OF SEVERE ACUTE MALNUTRITION MORTALITY RATE AMONG CHILDREN AGED 6-59 MONTHS ADMITTED AT PAWE GENERAL HOSPITAL STABILIZATION CENTER, NORTHWEST ETHIOPIA 2021,PLOS ONE*

| | Comment | Part | Correction | Amendment |
|---|---|---|---|---|
| #1 | "During SAM inpatient treatment recovery, transfer out, lost follow-up, and death was found 326 (57.39%), 106(18.66%), 46 (8.10%), and 91(16.02%), respectively.<br>The overall incidence of the mortality rate was 16.03 per 100 (95%CI: 13.86; 20.04) person-days observations<br>***"why you put two different rates for mortality"***<br>***"Operational words" with Definitions***" | **Abstract** | ***Accepted and corrected*** | Accepted & amended |
| #2 | "or mid-upper-arm circumference ≤115 mm, or presence of bilateral edema, and failed appetite test should be admitted for inpatient care[23].The current MUAC definition is <115 mm. Please change. The cited reference is fine. | **Methods** | ***Accepted and corrected*** | Amended<br>Look Page 6<br>Reference (23) |
| #3 | The Sphere cut off is <10% but <11% in the Abstract. Please correct. | **Abstract** | ***Accepted & incorporated*** | **Accepted**<br>**Look 2&11** |

| | | | | |
|---|---|---|---|---|
| **#4** | A majority usually means > 50% so please amend this sentence. Why not say that mortality in your series was high and half the deaths occurred within 2 weeks. Then list the key factors and suggest clinical care be focused preferentially on these risk factors | **Conclusio n** | **Accepted** | |
| **#5** | *Table 1. I cannot find the drug "Permaquinin" on Google or in the Ethiopian Guidelines for treating SAM. Please tell us the name of this drug.* | **Table 3** | It is cut out from interpretation, But you can read about Primaquine on<br><br>***For further more please read it or brows it***<br><br>*https://en.wikipedia.org/wiki/Primaquine* | **Amended** |
| **#6** | Table 2. Please explain what "Waiting time" means. | **Table 2** | ***Interpreted as hospital length of stay during treatment*** | **Amended** |
| **#7** | Table 4. This is unclear. Is it showing an overall survival rate for all the children? If so, 21.09% survival is much less than the reported survival in the paper:"At the end of this study period, 326 (57.39%) admitted under-five children had been cured | **Table 4** | **Removed & corrected and interpreted** | **Amended** |
| **#8** | Figures. Labelling of the Y axes of the 3 figures has been cut away. Please make sure we can see the labels. | | **It is re interpreted based on your request** | **Accepted and amended** |
| **#9** | Figure 3. This makes no sense to me. It shows a very low survival rate. I suggest you either remove this Figure or put in the correct KM curve. | **Figure 3** | ***Changed based on your queries and re- interpreted*** | **Amended** |
| **#10** | Figure 7. Analysis is not spelt correctly on the X-axis. | **Figure 7** | **Removed and re interpreted what you want to changed** | **Accepted and amended** |
| | | | | |

## *Reviewer reports:*

| **Reviewer #1:** | | **Area** | **Response of Authors** | **Status** |
|---|---|---|---|---|
| #1 | Abstract as grammatical errors | Abstract | We made the extensive grammatical correction and rephrasing with by authors and senior researcher consultation. | Accepted &Amended |
| #2 | Was the study conducted in one or many hospitals author said ' hospitals' | Title &Study area | The study conducted only one hospital i.e. Pawe General Hospital | Fully amended |
| #3 | Provide more details about the Stabilizing Center. Is this award treating both admission illness and nutrition deficiency or for SAM children? | Study area | -After SAM cases diagnosed in the pediatrics ward then transferred to stabilizing center<br>-which is isolated built for the only treatment of SAM cases and their complications<br>-Pediatrician and Trained health personnel were used supervised overall SC treatment based on national protocol | Amended % Incorporated |
| #4 | Understudy population, the study recruited children under management between 2008 and 2012. This is an approximately ten years old cohort. Is there a valid reason for not studying the latest cohort | Study period & Study population | -In fact, the SAM observation cohort was registration on a computer-based on Ethiopian colander (EC) which is late almost 7 years.<br>-However, we have changed and edited our mother document based on Gregorian calendar (GC) form;-<br>**-Marked corrected study periods and study population is as follows.**<br>**"**A retrospective cohort study was conducted in 568 under-five children who were admitted for SAM treatment at Pawe General Hospital ***from 1st January 2015 up to December 31, 2019***." | Accepted |
| #5 | Explain clearly the sample size estimation. What is a½ **=0.05?** What were the cases and non-exposed groups? | Samples size | We determined the minimum sample size by Survival sample size by STATA(SE)\14 sample size calculation<br>The sample size for the second objective was determined using STATA/SE version-14 by considering the following statistical assumptions two-sided significance level (α = 5%), Za/2 = Z value at 95% confidence | Accepted |

| | | | | |
|---|---|---|---|---|
| | | | interval = 1.96, power(**$Z_B$)=**80%, and P = % cumulative occurrence of death rate, 1.65 HR. Accordingly, a total of 440 SAM children's records were recruited.<br>*Final* Sample size was<br>$(n) = \frac{Event}{P\ (Event)} = \frac{(Z_{a/2}+Z_B)2}{\theta^2 p\ (1-p)}$ $n= \frac{e}{p\ (e)}$<br>$\theta$ = ln(HR)<br>HR= $e^{\theta}$ Where ZB = power of the study.<br>Za/2 = Z value at 95% confidence interval e = event<br>P (e) = probability of eventP =cumulative mortality rate from previous research *46%[6],* HR= hazard ratio from previous research (AHR) 1.6 [6] | |
| #6 | Again, the sample size estimated was 596, which is different from the numbers reported so far. | Sample size issue | We already calculated by single population proportion formula of sample size by using listed assumptions The final sample size was calculated as 578; | Amended |
| #7 | The inclusion criteria contradict what is explaining in the study population about the period of recruitment. Here it is mentioned children with SAM treated 2015-2019 were eligible | Inclusion criteria | The study period is changed and the inclusion criteria issue is resolved as following "All SAM admitted & treatment started children from January 1st, 2015, up to December 30, 2019, were considered as the study population and included" | Amended |
| #8 | **What** was the discharge criterion from the Stabilizing Center? | Outcome ascertainment | A child reached: weight-for-height/length is ≥–2 Z-score, and they have had no edema for at least 2<br>weeks, And/or MUAC (Mid-upper-arm circumference) is >115 mm and they have had no edema for at least 2 weeks.- If there are no danger signs for >15 min. | Fully amended |
| #9 | How were the z-scores calculated? | Methodolog | -It is the probability of a score occurring within a standard normal distribution curve: tells how many standard deviations from the mean your score.<br>-The formula for calculating a z-score is z = (x-μ)/σ, where x is the raw score, μ is the population mean, and σ is the population standard deviation.<br>-you can use the z-table and the normal distribution graph to give you a visual about how a z-score of 2.0 means "higher than average" | Accepted |
| #10 | -How was the survival analysis conducted?<br>-How was exposure time defined?<br>-Which statistical software has used Any testing of non- | Data analysis part | -Time-to-event (TTE) data is unique because the outcome of interest is not only whether or not an event occurred, but also when the event occurred.<br>-There needed 4 main methodological considerations, target event, the time origin, the time scale, and to describe how participants will exit | Accepted |

| | | | | |
|---|---|---|---|---|
| | informative censoring assumption | | -Time origin is the point at which follow-up time starts in the case of our study(January 1st, 2015-December 30 2019. we used STATA(SE) version 14 all survival analysis and assumption test<br>- Informative censoring is analogous to non-ignorable missing data, which will bias the analysis.<br>-There is no definitive way to test whether censoring is non-informative censoring is reasonable.<br>-In some instances proposed random sub-sample of censored subjects is followed to determine failure times when incomplete information is available about the survival time of SAM cases. | |
| #11 | Why were 568 children recruited out of the 596 eligible children? | Sample size issue | -We used for analysis 568 samples out of 578, and 10 participant cards were excluded due to treatment outcomes were not registered. | Corrected & incorporated |
| #12 | "457(80.46%) of admitted children were new admissions." What does this mean? | Result part | -Which means '457 cases were new admissions for stabilizing center?<br>-The remaining 111(19.54) cases were relapse SAM cases & Re-admitted twice within | No changed as it is |
| #13 | The authors reported the defaulted and recovered, these are new terms not defined in the methods | Operational words | We made correction and incorporated | Amended |
| #14 | The authors report "307 (54.35%) admitted children had at least one form of comorbidity" does this mean the remaining children were uncomplicated SAM?<br>Why would uncomplicated SAM be admitted to the hospital? | Result part | Thanks! this is an expression error<br>-All 568 SAM admitted children had manifold cases diagnosing and responsible for admission<br>-Of this 307 (54.35%) had more than two concomitant cases diagnosing and admitting for eg,<br>(commonly SAM with Pneumonia)+ anemia, or diarrhea or vomiting, etc. | Amended |
| #15 | How was anemia defined? | Operational | Anemia:-was defined and classified based on the mean amount of red blood cell or Hgb levels and classified into two ways:- No anemia >11 g/dL, and Anemia≤10.9 g/dl | Amended |
| *#16* | Were the 432 children managed with antimalarial have a positive slide for malaria? | Result report | *Read*<br>*https://reference.medscape.com/drug/primaquine-342691* | Amended |
| #17 | Provide the length of time (days) that children were on F75 and F100. | Result | SAM inpatient waiting in Stabilizing center was based on three phases of treatments.<br>**Phase -1=** In a separate room or space<br>-starter formula for inpatient management | Accepted |

| | | | | |
|---|---|---|---|---|
| | | | -Only F-75% formula milk used<br>-Weight is measured & plotted on the chart.<br>- Standard clinical signs (vomiting, dehydration, cough, respiration, and liver size were measure<br>- Amount of F-75 to give during Phase 1 is based on the class of weight (Kg).<br>-took2-6observation with clinical improvement consideration.<br>-**Transition phase**=<br>- Use only the F100 formula<br>-Daily surveillance of the child remains the same in Phase 1.<br>-F-100 milk given are based on the class of weight (Kg)<br>- Frequency of feeds should normally be 6 per day.<br>-Routine antibiotics also continued as necessary<br>- A maximum of 4-5 days with considerations of clinical improvement of cases<br>**Phase -2= Use** F-100 milk<br>**-**Every morning assessed temperature, vomiting, dehydration, respiration, etc.<br>-Determined by clinical improvement starting from 2 days of entry----). | |
| #18 | "In addition, more than half of the 326 (57.51%) and 104 (18.31%) cases were declared as cured and absent" What does absent mean? | Result & operation words | Look result part of Table-2:-<br>-After admitted and started SAM treatment Within five years cohort -326 (57.38%) cases had been cured, 91(16.73%) cases had died, **46(8.09)** cases- had been transferred out.**105(18.3**%)cases had <u>lost from follow-up</u> (Abscond) were the similar operational definition for Ethiopia FMOH SAM Guidelines.<br>You can read **https://motherchildnutrition.org/resources/pdf/mcn-ethiopia-sam-guidelines.pdf**. | Accept and changed |
| #19 | "The overall mortality incidence rate of severe acute malnutrition during the study period was found (IDR=16.73; 95%CI: 13.86--20.04)."Check the 95% CI correct. | Result part | -We repeatedly assessed what you requested to check the result of the incidence rate. Nevertheless, the result is what we had already reported and similar with –<br>-There is no result deviation both in STATA (SE) version R/14 software and in SPSS version 16. | Amended |
| #20 | "At the end of three weeks (29 days of follow-up) treatment" three weeks have 21days and not 29 days. Were children on follow-up for a fixed 3 weeks? | Result report | We corrected the grammatical errors | Accepted &Amended |
| #21 | -"Thus, within five years of risk observation, 473 children had censored results & gives 505 person-per-day observations (PDOs)."<br>-What does this mean? You have not explained who and when censoring was done | Result part | -We remove the term person per day of observation(PDOs) expression from the document<br>incorporated on the mother document<br><br>You can look at page 12, from line 8 | Accepted and incorporated |

| | | | | |
|---|---|---|---|---|
| #22 | Why are P-values reported as P≤ rather than the standard P<? | Predictors | We corrected and incorporated | Accepted and amended |
| #23 | -What was the rationale for including F-75 and F-100 in the regression models?-It does not make sense to include these in the models | Result part | We authors corrected and incorporated on the final model. | Accepted and amended |
| #24 | -How long did the children spend in the hospital? How long after admission did the deaths occur? Deaths in the first 3 days)and late deaths? | Result part | Maximum of 40 days but in our set up maximum 29 days please let me invited to see (Tables -4 ) Life tables | Amended |

**Part II= reviewer #2**

| Reviewer #2 comments | | Area of comment | Authors response | Status |
|---|---|---|---|---|
| #1 | -The introduction focuses on malnutrition broadly as well as SAM specifically, but sometimes it is unclear which of these definitions the authors are referring to.<br>-It might be beneficial if the authors mainly focus on SAM and the rationale for examining predictors of mortality in children with SAM. | Introduction | -We accepted & rephrasing part of the introduction part. It is attractive now<br>-Let me invited you to read and check it. | accepted & changed |
| #2 | This analysis used data from 2008 and the end of 2012. (However, later on, the inclusion criteria are stated between 2015-2019 so it is unclear what years were used.)If the admissions were between 2008 and 2012, what are the potential limitations of using data from several years ago, and why were more recent data not considered | Inclusion criteria | Thanks for point of view<br>-SAM treatment outcome registration in the facility was based on Ethiopian colander (EC)registration<br>, which is late almost -7 years from G.C.<br>-However, we have changed and edited all years based on the Gregorian calendar in our mother document-based<br><br>"A retrospective cohort study was conducted in 568 under-five children who were admitted for SAM treatment at Pawe General Hospital **from 1st January 2015 up to December 31, 2019."** | Accepted |
| #3 | The authors should also discuss changes in treatment protocols over time, as updates to the WHO | Operational words | -This is recommendations below were confirmed as current WHO to SAM | |

| | | | | |
|---|---|---|---|---|
| | guidelines were released in 2013 for example.<br><br>-It would also be important to explain the admission criteria for this treatment center | | management of children who are 6–59 months of age<br>-Weight-for-height ≤–3 Z-score, or<br>-Mid-upper-arm-circumference<115 mm, or presence of bilateral edema, & failed appetite test were admission criteria<br>-However, visible severe wasting is not included as a diagnostic criterion. | Accepted & incorporated |
| #4 | In the abstract and the introduction, the authors use the idiom "lion's share" which may not be familiar to all readers, so perhaps this could be changed to more common terminology and to specify whether this is about the absolute number of deaths or mortality in the comparison group | Abstract & Introduction | -We had changed the term "lions share" substituted by phrases "largest proportions" | Accepted and incorporated |
| #5 | : In the abstract, is also unclear what the authors mean by "not take in" the F-75/F-100 formula.<br>Do the authors mean feeding difficulties or loss of appetite, or that children were not provided these formulas? | Abstract | -We build another model and removed those due to confounding, | Accepted incorporated |
| #6 | The sample size calculation is unclear and appears that it may be appropriate for a prospective study rather than a retrospective analysis and explained. | Sample size | We determined the minimum sample size using a single population proportion formula [n =[(Za/2)2P (1-P)]/d2] by assuming 95% confidence level (Z a/2 = 1.96), margin of error 5% overall SAM mortality rate from previous research46%[6], and 15 % addition for missing/incomplete data. The final sample size was 442. However, when the five years multi-chart was reviewed, 578 records were there and all were considered for analysis. | |
| #7 | The terms kwashiorkor and marasmus should be replaced by edematous malnutrition and severe wasting, respectively | Result | We accepted and changed terms on result and discussion par.t | Accepted & changed |
| #8 | The authors describe the mean weights of children with SAM, yet it would be more useful to provide z-scores if possible since it is difficult to interpret weights alone. | Result | -We made, as it is mean weight as it is rather Z-score calculation for all is tedious readers | Not changed |
