# Supplementary material for: Incidence and predictors of severe acute malnutrition mortality in children aged 6–59 months admitted at Pawe general hospital, Northwest Ethiopia
Source: PLoS One. 2022 Feb 25;17(2):e0263236. doi: 10.1371/journal.pone.0263236 (PMC8880861; doi:10.1371/journal.pone.0263236)
Supplement: Attachment — Submitted filename: INCIDENCE AND PREDICTORS OF SEVERE ACUTE MALNUTRITION MORTALITY RATE AMONG CHILDRENS FROM 6.docx [file pone.0263236.s003.docx]

INCIDENCE AND PREDICTORS OF SEVERE ACUTE MALNUTRITION MORTALITY RATE AMONG CHILDRENS FROM 6-59 MONTHS IN STABILIZING CENTER AT PAWE GENERAL HOSPITALS, NORTHWEST, ETHIOPIA 2020.

Abstract

Grammatical errors

Background

Grammatical errors; Second paragraph, second line starts with a lower case letter

Methods

Mismatch in the study numbers, the abstract states they recruited 568 admitted children but, in the methods, (study design and area), 454 SAM-admitted children were recruited.

Was the study conducted in one or many hospitals? The authors state “The study was conducted in Pawe General Hospitals”

Provide more details about the Stabilizing Center. Is this a ward treating both admission illness and nutrition deficient or it is holding centre for children being managed for SAM only?

Under study population, the study recruited children under management between 2008 and 2012. This is approximately ten years old cohort. Is there a valid reason for not studying latest cohort?

Please explain clearly the sample size estimation. What is a ½ =0.05? What were the cases and non-exposed group?

Again, the sample size estimated was 596 which is different from numbers reported so far.

Under independent variables, what are WFA, WFH, HFA?

The inclusion criteria contradict what is explain in the study population about the period of recruitment. Here it is mentioned children with SAM treated 2015-2019 were eligible.

What was the discharge criteria from the Stabilizing Center?

Does cured mean a child who had resolved SAM at discharge?

How were the z-scores calculated?

How was the survival analysis conducted? Which statistical software was used? How were exposure time defined? Were any children censored? Any testing of non-informative censoring assumption?

Results

Why were on 568 children recruited out of the 596 eligible children?

“457(80.46%) of admitted children were new admissions.” What does this mean?

The authors reported the defaulted and recovered, these are new terms not defined in the methods.

The authors report “Three hundred and seven 307 (54.35%) admitted children had at least one form of comorbidity” does this mean the remaining children were uncomplicated SAM? Why would uncomplicated SAM be admitted in hospital?

How was anemia defined?

Were the 432 children managed with antimalaria have a positive slide for malaria?

Provide the length of time (days) that children were on F75 and F100.

“In addition, more than half of the 321 (56.51%) and 104 (18.31%) cases were declared as cured and absent” What does absent mean?

“The overall mortality incidence rate of severe acute malnutrition during the study period was found (IDR=16.73; 95%CI: 13.8620.04).” Check the 95% CI, it is not correct.

“At the end of three weeks (29 days of follow-up) treatment” three weeks has 21days and not 29 days. Were children on follow-up for a fixed 3 weeks?

“Thus, within five years of risk observation, 473 children had censored results and produced 3794 person-per-day observations (PDOs).” What does this mean? You have not explained who and when censoring was done.

Why are P-values reported as P≤ rather than the standard P<?

What was the rationale of including F75 and F100 in the regression models? These are standard treatment for SAM. F100 is started after stabilising when most deaths could have occurred. It doesn’t make sense to include these in the models.

How long did the children spend in the hospital? How long after admission did the deaths occur? Was there difference between early deaths (deaths in first 3 days) and late deaths?
