# Supplementary material for: Incidence and predictors of severe acute malnutrition mortality in children aged 6–59 months admitted at Pawe general hospital, Northwest Ethiopia
Source: PLoS One. 2022 Feb 25;17(2):e0263236. doi: 10.1371/journal.pone.0263236 (PMC8880861; doi:10.1371/journal.pone.0263236)
Supplement: Attachment — Submitted filename: Response to Reviewers .docx [file pone.0263236.s004.docx]

**Author’s response to reviews**

**Titles:-INCIDENCE AND PREDICTORS OF SEVERE ACUTE MALNUTRITION MORTALITY RATE AMONG 6-59 MONTHS CHILDREN IN PAWE GENERAL HOSPITAL NORTHWEST ETHIOPIA 2021**

**Authors email**

1) **Fassikaw Kebede:-**[**fassikaw123@gmail.com**](mailto:fassikaw123@gmail.com)
2) **Belete Negese: -** [**yabebalij@gmail.com**](mailto:yabebalij@gmail.com)
3) **Ayalew Kassaw: -** [**ayalewkassaw25@gmail.com**](mailto:ayalewkassaw25@gmail.com)
4) **Atitegeb Abera:-**[**atitegebabera@gmail.com**](mailto:atitegebabera@gmail.com)
5) **Getahun Fentaw:-**[**gechfentaw1014@gmail.com**](mailto:gechfentaw1014@gmail.com)

- **Version1: Date 16 June 2021**
- **Author response for reviewers**

**Reviewer reports:**

| **Reviewer #1:** | | Area of comment | Response of Authors | Status |
| --- | --- | --- | --- | --- |
| #1 | Abstract as grammatical errors | Abstract | We made extensive grammatical correction and rephrasing with by authors and senior researcher consultation. | Partially accepted &Amended |
| #2 | Was the study conducted in one or many hospitals author said ‘ hospitals’ | Title &Study area | The study conducted only one hospital i.e. Pawe General Hospital | Fully amended |
| #3 | Provide more details about the Stabilizing Center. Is this award treating both admission illness and nutrition deficiency or for SAM children? | Study area | -After SAM cases diagnosed in the pediatrics ward then transferred to stabilizing center  -which is isolated built for the only treatment of SAM cases and their complications  -Pediatrician and Trained health personnel were used supervised overall SC treatment based on national protocol | Fully amended  Incorporated |
| #4 | Understudy population, the study recruited children under management between 2008 and 2012.  This is an approximately ten years old cohort. Is there a valid reason for not studying the latest cohort | Study period &  Study population | -In fact, the SAM observation cohort was registration on a computer-based on Ethiopian colander (EC) which is late almost 7 years.  -However, we have changed and edited our mother document based on Gregorian calendar (GC) form;-  -**Marked corrected study periods and study population is as follows.**  **“**A retrospective cohort study was conducted in 568 under-five children who were admitted for SAM treatment at Pawe General Hospital ***from 1^st^ January 2015 up to December 31, 2019***.” | Fully changed & Incorporated |
| #5 | Explain clearly the sample size estimation. What is a½ **=0.05?** What were the cases and non-exposed group? | Samples size | **-The** sample size of this study was calculated based  on the double population proportion formula using Epi-info version 7 by considering the following assumptions.  -Two-sided significant level (α) of 5%, Z**a½** value at 95%CI=1.96, power 80%, death rate= 29.2% , Hazard Ratio (AHR)=1.56--,used & software gives 527.  -Adding 10%, adjustment for the incompleteness of secondary data files& finally sample size would be as 578. | Accepted & substituted by software-based |
| #6 | Again, the sample size estimated was 596, which is different from the numbers reported so far. | Sample size issue | -We already calculated by double population proportion formula of sample size by using listed assumptions  -The final sample size was calculated as 578; | Fully amended |
| #7 | The inclusion criteria contradict what is explaining in the study population about the period of recruitment. Here it is mentioned children with SAM treated 2015-2019 were eligible | Inclusion criteria | The study period is changed and the inclusion criteria issue is resolved as following "All SAM admitted & treatment started children from January 1^st^, 2015, up to December 30, 2019, were considered as the study population and included” | Partially changed |
| #8 | **What** was the discharge criterion from the Stabilizing Center? | Outcome ascertainment | After hospitalization SAM treatment a maximum of 40 days discharged when:-  A. children reached:weight-for-height/length is ≥–2 Z-score, and they have had no edema for at least 2  weeks,  -And/or MUAC (Mid-upper-arm circumference) is ≥115 mm and they have had no edema for at least 2 weeks.  - If there are no danger signs for >15 min. | Fully amended |
| #9 | How were the z-scores calculated? | Methodology | -It is the probability of a score occurring within a standard normal distribution curve: tells how many standard deviations from the mean your score.  -The formula for calculating a z-score is z = (x-μ)/σ, where x is the raw score, μ is the population mean, and σ is the population standard deviation.  -you can use the z-table and the normal distribution graph to give you a visual about how a z-score of 2.0 means “higher than average” | Accepted |
| #10 | -How was the survival analysis conducted?  -How was exposure time defined?  -Which statistical software was used  -Any testing of non-informative censoring assumption | Data analysis part | -Time-to-event (TTE) data is unique because the outcome of interest is not only whether or not an event occurred, but also when the event occurred.  -There needed 4 main methodological considerations, target event, the time origin, the time scale, and to describe how participants will exit  -Time origin is the point at which follow-up time starts in the case of our study(January 1^st^, 2015-December 30 2019  -we used STATA(SE) version 14 all survival analysis and assumption test  - Informative censoring is analogous to non-ignorable missing data, which will bias the analysis.  -There is no definitive way to test whether censoring is non-informative censoring is reasonable.  - In some instances proposed random sub-sample of censored subjects is followed to determine failure times when incomplete information is available about the survival time of SAM cases. | Accepted |
| #11 | Why were 568 children recruited out of the 596 eligible children? | Sample size issue | -We used for analysis 568 samples out of 578, and 10 participant cards were excluded due to treatment outcome were not registered. | Corrected & incorporated |
| #12 | **“**457(80.46%) of admitted children were new admissions.” What does this mean? | Result part | -Which means ‘457 cases were new admissions for stabilizing center?  -The remaining 111(19.54) cases were relapse SAM cases & Re-admitted twice within | No changed as it is |
| #13 | The authors reported the defaulted and recovered, these are new terms not defined in the methods | Operational words | Thanks  we included on methods part | Amended and incorporated |
| #14 | The authors report “307 (54.35%) admitted children had at least one form of comorbidity” does this mean the remaining children were uncomplicated SAM?  Why would uncomplicated SAM be admitted to the hospital? | Result part | Thanks! this is an expression error  -All 568 SAM admitted children had manifold cases diagnosing and responsible for admission  -Of this 307 (54.35%) had more than two concomitant cases diagnosing and admitting for eg,  (commonly SAM with Pneumonia)+ anemia, or diarrhea or vomiting, etc. | Amended but no changed |
| #15 | How was anemia defined? | Operational | Anemia was defined and classified based on cell  size: Mean amount of red blood cell or Hgb levels and classified into three ways in pediatrics  1). Mild anemia: 10-10.9 g/dL  2. Moderate anemia: 7-9.9 g/dL  3. Severe anemia: <7 g/dL  However in our set up we only classified two categories 1) <11gm/dl 2) >=11 g/dl |  |
| *#16* | Were the 432 children managed with antimalarial have a positive slide for malaria? | Result report | *-*Ethiopian (FMOH) manuals for SAM of infant & children ordered for all admitted cases,**pre-maquinien anti-malaria prophylaxis’s** after the screening of blood film as a protocol in Stabilizing center.  - By default study area had a repeated malaria outbreak history  Therefore from 568 studied participants, 432 cases had got a pre-maquinien anti-malaria prophylaxis after negative testing,  -But the remaining 136 cases treated by anti-malaria after positive slide checking. Read for further information[**https://motherchildnutrition.org/resources/pdf/mcn-ethiopia-sam-guidelines.pdf**](https://motherchildnutrition.org/resources/pdf/mcn-ethiopia-sam-guidelines.pdf) | Not changed |
| #17 | Provide the length of time (days) that children were on F75 and F100. | Result | SAM inpatient waiting in Stabilizing center was based on three phases of treatments .  **Phase -1=** In a separate room or space  -starter formula for inpatient management  -Only F-75% formula milk used  -Weight is measured & plotted on the chart .  - Standard clinical signs (vomiting, dehydration, cough, respiration, and liver size were measure  - Amount of F-75 to give during Phase 1 is based on the class of weight (Kg).  -A maximum 2-8 days with consideration of clinical improvement.  -**Transition phase**=  - Use only the [F100 formula](https://motherchildnutrition.org/malnutrition-management/info/feeding-formulas-f75-f100.html)  -Daily surveillance of the child remains the same in Phase 1.  -F-100 milk given are based on the class of weight (Kg)  - Frequency of feeds should normally be 6 per day.  -Routine antibiotics  also continued as necessary  - A maximum of 4-5 days with considerations of clinical improvement of cases  **Phase -2= Use** [F-100](https://motherchildnutrition.org/malnutrition-management/info/feeding-formulas-f75-f100.html) milk  **-**Every morning assessed temperature, vomiting, dehydration, respiration, etc.  -Determined by clinical improvement starting from 2 days of entry----). | Accepted &incorporated |
| #18 | “In addition, more than half of the 326 (57.51%) and 104 (18.31%) cases were declared as cured and absent” What does absent mean? | Result & operation words | Look result part of Table-2:-  -After admitted and started SAM treatment Within five years cohort  -326 (57.38%) cases had cured,  - 91(16.73%) cases had died,  -*-* **46(8.09)** case- had transferred out.  **- 105(18.3**%)cases had lost from follow-up (Abscond) were the similar operational definition for Ethiopia FMOH SAM Guidelines.  You can read  [**https://motherchildnutrition.org/resources/pdf/mcn-ethiopia-sam-guidelines.pdf**](https://motherchildnutrition.org/resources/pdf/mcn-ethiopia-sam-guidelines.pdf). | Accept and changed |
| #19 | “The overall mortality incidence rate of severe acute malnutrition during the study period was found (IDR=16.73; 95%CI: 13.86--20.04).”  - Check the 95% CI, it is not correct. | Result part | -We had repeatedly assessed what you requested to check the result of mortality incidence.  Nevertheless, the result is what we had already reported and similar with –  -There is no result deviation both in STATA (SE) version R/14 software and in SPSS version 16. | We have checked  Amendment As it is |
| #20 | “At the end of three weeks (29 days of follow-up) treatment" three weeks have 21days and not 29 days. Were children on follow-up for a fixed 3 weeks? | Result report | We corrected the grammatical errors | Accepted &Amended |
| #21 | -“Thus, within five years of risk observation, 473 children had censored results & gives 505 person-per-day observations (PDOs).”  -What does this mean? You have not explained who and when censoring was done | Result part | -We remove the term person per day of observation(PDOs) expression from the document  incorporated on the mother document  You can look at page 12, from line 8 | Accepted and incorporated |
| #22 | Why are P-values reported as P≤ rather than the standard P<? | Predictors | We made the correction and incorporated | Accepted and amended |
| #23 | -What was the rationale for including F-75 and F-100 in the regression models?-It does not make sense to include these in the models | Result part | We authors corrected and incorporated on the final model. | Accepted and amended |
| #24 | -How long did the children spend in the hospital?  -How long after admission did the deaths occur? Deaths in the first 3 days)and late deaths? | Result part | Maximum of 40 days but in our set up maximum 29 day  Please  Let me invited to see (Tables -4 ) Life tables | Amended |

**Part II= reviewer #2**

| Reviewer #2 comments | | Area of comment | Authors response | Status |
| --- | --- | --- | --- | --- |
| #1 | -The introduction focuses on malnutrition broadly as well as SAM specifically, but sometimes it is unclear which of these definitions the authors are referring.  -It might be beneficial if the authors mainly focus on SAM and the rationale for examining predictors of mortality in children with SAM. | Introduction | -We accepted & rephrasing part of the introduction partially. It is attractive now  -Let me invited you to read and check it. | accepted & changed |
| #2 | This analysis used data from 2008 and the end of 2012. (However, later on, the inclusion criteria are stated between 2015-2019 so it is unclear what years were used.)If the admissions were between 2008 and 2012, what are the potential limitations of using data from several years ago, and why were more recent data not considered | Inclusion criteria | Thanks for point of view  -SAM treatment outcome registration in the facility was based on Ethiopian colander (EC)registration  , which is late almost -7 years from G.C.  -However, we have changed and edited totally all years based on Gregorian calendar in our mother document based  **“**A retrospective cohort study was conducted in 568 under five children who were admitted for SAM treatment at Pawe General Hospital **from 1^st^ January 2015 up to December 31, 2019.”** | Accepted |
| #3 | The authors should also discuss changes in treatment protocols over time, as updates to the WHO guidelines were released in 2013 for example.  -It would also be important to explain the admission criteria for this treatment center | Operational words | -This is recommendations below were confirmed as current WHO to SAM management of children who are 6–59 months of age  -Weight-for-height ≤–3 Z-score, or  -Mid-upper-arm-circumference<115 mm, or presence of bilateral edema, & failed appetite test were admission criteria  -However, visible severe wasting is not included as a diagnostic criterion. | Accepted & incorporated |
| #4 | In the abstract and the introduction, the authors use the idiom "lion's share" which may not be familiar to all readers, so perhaps this could be changed to more common terminology and to specify whether this is about the absolute number of deaths or mortality in the comparison group | Abstract & Introduction | -We had changed the term “lions share” substituted by phrases “largest proportions” | Accepted and incorporated |
| #5 | : In the abstract, is also unclear what the authors mean by "not take in" the F-75/F-100 formula.  Do the authors mean feeding difficulties or loss of appetite, or that children were not provided these formulas? | Abstract & result part | -We build another models and removed those due to confounding, | Accepted incorporated |
| #6 | The sample size calculation is unclear and appears that it may be appropriate for a prospective study rather than a retrospective analysis and explained. | Sample size | **-**Sample size of this study was calculated based on the double population proportion formula using Epi-info version 7computer program using the following assumptions.  -Two-sided significant level (α) of 5%, Z**a½** value at 95% confidence interval =1.96, power 80%,death rate= 29.2%,Hazard Ratio (AHR)from =1.56  -The Software gives =527& 10%, adjustment for secondary data files incompleteness and  -Final sample size would be=578. |  |
| #7 | The terms kwashiorkor and marasmus should be replaced by edematous malnutrition and severe wasting, respectively | Result part | We accepted and changed terms on result and discussion par.t | Accepted & changed |
| #8 | The authors describe the mean weights of children with SAM, yet it would be more useful to provide z-scores if possible since it is difficult to interpret weights alone. | Result part | -We made, as it is mean weight as it is rather Z-score calculation for all is tedious readers | Not changed |
