# Supplementary material for: Incidence and predictors of severe acute malnutrition mortality in children aged 6–59 months admitted at Pawe general hospital, Northwest Ethiopia
Source: PLoS One. 2022 Feb 25;17(2):e0263236. doi: 10.1371/journal.pone.0263236 (PMC8880861; doi:10.1371/journal.pone.0263236)
Supplement: Attachment — Submitted filename: Response to Reviewers .edited.docx [file pone.0263236.s005.docx]

**Author’s response to reviews**

**Titles:-INCIDENCE AND PREDICTORS OF SEVERE ACUTE MALNUTRITION MORTALITY RATE AMONG 6-59 MONTHS CHILDREN IN PAWE GENERAL HOSPITAL NORTHWEST ETHIOPIA 2021**

**Authors email**

1) **Fassikaw Kebede:-**[**fassikaw123@gmail.com**](mailto:fassikaw123@gmail.com)
2) **Belete Negese: -** [**yabebalij@gmail.com**](mailto:yabebalij@gmail.com)
3) **Ayalew Kassaw: -** [**ayalewkassaw25@gmail.com**](mailto:ayalewkassaw25@gmail.com)
4) **Atitegeb Abera:-**[**atitegebabera@gmail.com**](mailto:atitegebabera@gmail.com)
5) **Getahun Fentaw:-**[**gechfentaw1014@gmail.com**](mailto:gechfentaw1014@gmail.com)

**Editors comment for Author**

| **Academic Editors comment** | | **Area of comment** | Response of Authors | **Status** |
| --- | --- | --- | --- | --- |
| **#1** | Replace lion’s share with most | Abstract | Accept and incorporated | Changed |
| **#2** | paw – is Pawe hospital, please correct | Study area | Accepted and incorporated | changed |
| **#3** | Anaemia is defined as Hb < 11 g/dL in the text but it is < 10 g/dL in the Abstract. Please correct | **Operational definition** | Anemia:-was defined and classified based on the mean amount of red blood cell or Hgb levels and classified into two ways:- No anemia >11 g/dL, and Anemia≤10.9 g/dl | **Accepted** |
| **#4** | **Not clear about the percentages of beds – 29% and 25% are stated.** | **Study area** | Pawe general hospital had more than 252 beds for inpatient treatment. Stabilizing Center is separately built from pediatrics wards & having 27 single beds for only SAM treatments. Trained health professionals comprising (two resident physicians, Pediatrician case management, and nurse) were there in SC. | **accepted** |
| **#5** | Independent variables The terms kwashiorkor and marasmus should be replaced by edematous malnutrition and severe wasting, respectively | **Variables** | Accepted and changed | **accepted** |
| **#6** | Define all abbreviations like ARTI WFA etc |  | Accepted | Accepted |
| **#7** | MUAC definitions vary from < 115cm to <=115 cm. Please correct this. | **Admission criteria** | Accepted | **Accepted** |
| **#8** | The inclusion and exclusion criteria are the same | **Operation words** | Has accepted and incorporated | **Accepted** |
| **#9** | The dependent variable is not clear when death has to take place – is it as an inpatient in the stabilizing center or sometime after discharge. | **Methodology** | Mortality of SAM admitted children (Death is an event of interest /cured is censored observation). | **Accepted** |
| **#10** | comorbidity One in 5 = 20% not 11.98%. Please just report the %s and numerators and denominators Remove, etc | **Result part** | Accepted and incorporated | **Accepted** |
| **#11** | Antimalarial treatment i.e. short course or was it antimalarial prophylaxis? Please state which drugs were used and for how long. | **Result part** | In our study, 432(76.06%) admitted children were negative for malaria screening. However, the remaining 136(23.9%) cases were addresses both antimalarial and 0.25mg/kg **Premaquinin** for Plasmodium vivax but the stat is given for plasmodium Falciparum for eradication of gametocyte malaria from the liver for 14 consecutive days after confirmed For further information let me invite to read it[**https://motherchildnutrition.org/resources/pdf/mcn-ethiopia-sam-guidelines.pdf**](https://motherchildnutrition.org/resources/pdf/mcn-ethiopia-sam-guidelines.pdf) | **Accepted**  **Remove ambition** |
| **#12** | Please tell us what is in F75and F100 and what the indications are to give one or the other | **Methods** | F75 (130ml = 100kcal) milk therapeutic food was used during phase I treatment. During transferred to transition phase to receive F100 (diluted F100, 130ml = 130kcal), F100 diluted is given at transition phase.  In phase II treatment, F100 was used as much as the children could take and additional diet is recommended until they achieved weight for height *>* 85% and no edema for 10 consecutive day | **Accepted** |
| **#13** | Mortality incidence I do not understand where the median follow-up period of 13.5 days comes from. Please tell us how long children were admitted in the SC and how long the follow-up period was after discharge | **Discussion and result part** | This is not to say median but average (mean) waiting for the time of Hospitalization based on Ethiopian SPHERE reference 13.5 days in this study agrees with | **Accepted** |
| **#14** | References The text has a reference of 29 but the reference list stops at 20. | **Reference** | Dear editor realty it is not 29 but it is r 39 reference and let invite you to see | No changed |
| **#15** | Repetitive text: Descriptive analyses such as tables, graphs, Kaplan–Meier survival curves, and the log-rank test were | Data analysis and processing part | We corrected and incorporated | **Accepted** |
|  | ***Via main Author Email sent***  I sought independent statistical advice on your sample size calculation after the letter was sent to Plos One. Sample size of this study was calculated based on double population proportion formula of using Epi info version 7computer program by considering the following assumptions, Two-sided significant level (α) of 5%, power 80%, Z a½ value at 95% confidence interval = 1.96, death rate= 29.2%, Adjusted Hazard Ratio (AHR)= 1.56[15], was used to calculate 527   He says “This doesn't make sense. They should rewrite.” | Sample size  Should to rewrite | We determined the minimum sample size using a both single population proportion for first objective and Survival sample size by STATA(SE)\14 sample size calculation  1)single population proportion formula [n =[(Za/2)2P (1-P)]/d2] by assuming 95% confidence level (Z a/2 = 1.96), margin of error 5% overall SAM mortality rate from previous research46%[6], and 15 % addition for missing/incomplete data. The final sample size was 442. However, when the five years multi-chart was reviewed, 578 records were there and all are included for analysis  The sample size for the second objective was determined using STATA/SE version-14 by considering the following statistical assumptions two-sided significance level (α = 5%), Za/2 = Z value at 95% confidence interval = 1.96, power(**Z_B_)=**80%, and P = % cumulative occurrence of death rate, 1.65 HR. Accordingly, a total of 440 SAM children’s records were recruited.  *Final* Sample size was  (n) =Event = **(Z _a/2_+Z_B_)2 n= e**  P (Event) **θ ^2^p (1-p) p (e)**  *θ* = ln(HR)  HR= e*^θ^*  Where  ZB = power of the study.  Za/2 = Z value at 95% confidence interval  e = event  P (e) = probability of event  P =cumulative mortality rate from previous research *46%[6],*  HR= hazard ratio from previous research (AHR) 1.6 [6]  *.* | **Accepted** |

**Reviewer reports:**

| **Reviewer #1:** | | **Area** | **Response of Authors** | **Status** |
| --- | --- | --- | --- | --- |
| #1 | Abstract as grammatical errors | Abstract | We made the extensive grammatical correction and rephrasing with by authors and senior researcher consultation. | Accepted &Amended |
| #2 | Was the study conducted in one or many hospitals author said ‘ hospitals’ | Title &Study area | The study conducted only one hospital i.e. Pawe General Hospital | Fully amended |
| #3 | Provide more details about the Stabilizing Center. Is this award treating both admission illness and nutrition deficiency or for SAM children? | Study area | -After SAM cases diagnosed in the pediatrics ward then transferred to stabilizing center  -which is isolated built for the only treatment of SAM cases and their complications  -Pediatrician and Trained health personnel were used supervised overall SC treatment based on national protocol | Amended %  Incorporated |
| #4 | Understudy population, the study recruited children under management between 2008 and 2012. This is an approximately ten years old cohort. Is there a valid reason for not studying the latest cohort | Study period &  Study population | -In fact, the SAM observation cohort was registration on a computer-based on Ethiopian colander (EC) which is late almost 7 years.  -However, we have changed and edited our mother document based on Gregorian calendar (GC) form;-  -**Marked corrected study periods and study population is as follows.**  **“**A retrospective cohort study was conducted in 568 under-five children who were admitted for SAM treatment at Pawe General Hospital ***from 1^st^ January 2015 up to December 31, 2019***.” | Accepted |
| #5 | Explain clearly the sample size estimation. What is a½ **=0.05?** What were the cases and non-exposed groups? | Samples size | We determined the minimum sample size using a both single population proportion for first objective and Survival sample size by STATA(SE)\14 sample size calculation  1)single population proportion formula [n =[(Za/2)2P (1-P)]/d2] by assuming 95% confidence level (Z a/2 = 1.96), margin of error 5% overall SAM mortality rate from previous research46%[6], and 15 % addition for missing/incomplete data. The final sample size was 442. However, when the five years multi-chart was reviewed, 578 records were there and all are included for analysis  The sample size for the second objective was determined using STATA/SE version-14 by considering the following statistical assumptions two-sided significance level (α = 5%), Za/2 = Z value at 95% confidence interval = 1.96, power(**Z_B_)=**80%, and P = % cumulative occurrence of death rate, 1.65 HR. Accordingly, a total of 440 SAM children’s records were recruited.  *Final* Sample size was  (n) =Event = **(Z _a/2_+Z_B_)2 n= e**  P (Event) **θ ^2^p (1-p) p (e)**  *θ* = ln(HR)  HR= e*^θ^* Where ZB = power of the study.  Za/2 = Z value at 95% confidence interval e = event  P (e) = probability of eventP =cumulative mortality rate from previous research *46%[6],* HR= hazard ratio from previous research (AHR) 1.6 [6] | Accepted & substituted by software-based |
| #6 | Again, the sample size estimated was 596, which is different from the numbers reported so far. | Sample size issue | -We already calculated by single population proportion formula of sample size by using listed assumptions The final sample size was calculated as 578; | Fully amended |
| #7 | The inclusion criteria contradict what is explaining in the study population about the period of recruitment. Here it is mentioned children with SAM treated 2015-2019 were eligible | Inclusion criteria | The study period is changed and the inclusion criteria issue is resolved as following "All SAM admitted & treatment started children from January 1^st^, 2015, up to December 30, 2019, were considered as the study population and included” | Partially changed |
| #8 | **What** was the discharge criterion from the Stabilizing Center? | Outcome ascertainment | A child reached: weight-for-height/length is ≥–2 Z-score, and they have had no edema for at least 2  weeks, And/or MUAC (Mid-upper-arm circumference) is >115 mm and they have had no edema for at least 2 weeks.- If there are no danger signs for >15 min. | Fully amended |
| #9 | How were the z-scores calculated? | Methodology | -It is the probability of a score occurring within a standard normal distribution curve: tells how many standard deviations from the mean your score.  -The formula for calculating a z-score is z = (x-μ)/σ, where x is the raw score, μ is the population mean, and σ is the population standard deviation.  -you can use the z-table and the normal distribution graph to give you a visual about how a z-score of 2.0 means “higher than average” | Accepted |
| #10 | -How was the survival analysis conducted?  -How was exposure time defined?  -Which statistical software has used Any testing of non-informative censoring assumption | Data analysis part | -Time-to-event (TTE) data is unique because the outcome of interest is not only whether or not an event occurred, but also when the event occurred.  -There needed 4 main methodological considerations, target event, the time origin, the time scale, and to describe how participants will exit  -Time origin is the point at which follow-up time starts in the case of our study(January 1^st^, 2015-December 30 2019. we used STATA(SE) version 14 all survival analysis and assumption test  - Informative censoring is analogous to non-ignorable missing data, which will bias the analysis.  -There is no definitive way to test whether censoring is non-informative censoring is reasonable.  -In some instances proposed random sub-sample of censored subjects is followed to determine failure times when incomplete information is available about the survival time of SAM cases. | Accepted |
| #11 | Why were 568 children recruited out of the 596 eligible children? | Sample size issue | -We used for analysis 568 samples out of 578, and 10 participant cards were excluded due to treatment outcomes were not registered. | Corrected & incorporated |
| #12 | **“**457(80.46%) of admitted children were new admissions.” What does this mean? | Result part | -Which means ‘457 cases were new admissions for stabilizing center?  -The remaining 111(19.54) cases were relapse SAM cases & Re-admitted twice within | No changed as it is |
| #13 | The authors reported the defaulted and recovered, these are new terms not defined in the methods | Operational words | We incorporated | Amended and incorporated |
| #14 | The authors report “307 (54.35%) admitted children had at least one form of comorbidity” does this mean the remaining children were uncomplicated SAM?  Why would uncomplicated SAM be admitted to the hospital? | Result part | Thanks! this is an expression error  -All 568 SAM admitted children had manifold cases diagnosing and responsible for admission  -Of this 307 (54.35%) had more than two concomitant cases diagnosing and admitting for eg,  (commonly SAM with Pneumonia)+ anemia, or diarrhea or vomiting, etc. | Amended but no changed |
| #15 | How was anemia defined? | Operational | Anemia:-was defined and classified based on the mean amount of red blood cell or Hgb levels and classified into two ways:- No anemia >11 g/dL, and Anemia≤10.9 g/dl |  |
| *#16* | Were the 432 children managed with antimalarial have a positive slide for malaria? | Result report | *sorry for it!* In our study, 432(76.06%) admitted children were negative for malaria screening. However, the remaining 136(23.9%) cases were addresses both antimalarial and 0.25mg/kg ***Premaquinin*** *for 14 consecutive days or Stat dose confirmed for both* ***Plasmodium Vivax and Falciparum respectively*** *for eradication of gametocyte* let me invite you to read it [**https://motherchildnutrition.org/resources/pdf/mcn-ethiopia-sam-guidelines.pdf**](https://motherchildnutrition.org/resources/pdf/mcn-ethiopia-sam-guidelines.pdf) | Not changed |
| #17 | Provide the length of time (days) that children were on F75 and F100. | Result | SAM inpatient waiting in Stabilizing center was based on three phases of treatments.  **Phase -1=** In a separate room or space  -starter formula for inpatient management  -Only F-75% formula milk used  -Weight is measured & plotted on the chart.  - Standard clinical signs (vomiting, dehydration, cough, respiration, and liver size were measure  - Amount of F-75 to give during Phase 1 is based on the class of weight (Kg).  -took2-6observation with clinical improvement consideration.  -**Transition phase**=  - Use only the [F100 formula](https://motherchildnutrition.org/malnutrition-management/info/feeding-formulas-f75-f100.html)  -Daily surveillance of the child remains the same in Phase 1.  -F-100 milk given are based on the class of weight (Kg)  - Frequency of feeds should normally be 6 per day.  -Routine antibiotics  also continued as necessary  - A maximum of 4-5 days with considerations of clinical improvement of cases  **Phase -2= Use** [F-100](https://motherchildnutrition.org/malnutrition-management/info/feeding-formulas-f75-f100.html) milk  **-**Every morning assessed temperature, vomiting, dehydration, respiration, etc.  -Determined by clinical improvement starting from 2 days of entry----). | Accepted &incorporated |
| #18 | “In addition, more than half of the 326 (57.51%) and 104 (18.31%) cases were declared as cured and absent” What does absent mean? | Result & operation words | Look result part of Table-2:-  -After admitted and started SAM treatment Within five years cohort -326 (57.38%) cases had been cured, 91(16.73%) cases had died, **46(8.09)** cases- had been transferred out.  **- 105(18.3**%)cases had lost from follow-up (Abscond) were the similar operational definition for Ethiopia FMOH SAM Guidelines.  You can read  [**https://motherchildnutrition.org/resources/pdf/mcn-ethiopia-sam-guidelines.pdf**](https://motherchildnutrition.org/resources/pdf/mcn-ethiopia-sam-guidelines.pdf). | Accept and changed |
| #19 | “The overall mortality incidence rate of severe acute malnutrition during the study period was found (IDR=16.73; 95%CI: 13.86--20.04).”Check the 95% CI correct. | Result part | -We repeatedly assessed what you requested to check the result of the incidence rate. Nevertheless, the result is what we had already reported and similar with –  -There is no result deviation both in STATA (SE) version R/14 software and in SPSS version 16. | Amended |
| #20 | “At the end of three weeks (29 days of follow-up) treatment" three weeks have 21days and not 29 days. Were children on follow-up for a fixed 3 weeks? | Result report | We corrected the grammatical errors | Accepted &Amended |
| #21 | -“Thus, within five years of risk observation, 473 children had censored results & gives 505 person-per-day observations (PDOs).”  -What does this mean? You have not explained who and when censoring was done | Result part | -We remove the term person per day of observation(PDOs) expression from the document  incorporated on the mother document  You can look at page 12, from line 8 | Accepted and incorporated |
| #22 | Why are P-values reported as P≤ rather than the standard P<? | Predictors | We corrected and incorporated | Accepted and amended |
| #23 | -What was the rationale for including F-75 and F-100 in the regression models?-It does not make sense to include these in the models | Result part | We authors corrected and incorporated on the final model. | Accepted and amended |
| #24 | -How long did the children spend in the hospital? How long after admission did the deaths occur? Deaths in the first 3 days)and late deaths? | Result part | Maximum of 40 days but in our set up maximum 29 days please let me invited to see (Tables -4 ) Life tables | Amended |

**Part II= reviewer #2**

| Reviewer #2 comments | | Area of comment | Authors response | Status |
| --- | --- | --- | --- | --- |
| #1 | -The introduction focuses on malnutrition broadly as well as SAM specifically, but sometimes it is unclear which of these definitions the authors are referring to.  -It might be beneficial if the authors mainly focus on SAM and the rationale for examining predictors of mortality in children with SAM. | Introduction | -We accepted & rephrasing part of the introduction part. It is attractive now  -Let me invited you to read and check it. | accepted & changed |
| #2 | This analysis used data from 2008 and the end of 2012. (However, later on, the inclusion criteria are stated between 2015-2019 so it is unclear what years were used.)If the admissions were between 2008 and 2012, what are the potential limitations of using data from several years ago, and why were more recent data not considered | Inclusion criteria | Thanks for point of view  -SAM treatment outcome registration in the facility was based on Ethiopian colander (EC)registration  , which is late almost -7 years from G.C.  -However, we have changed and edited all years based on the Gregorian calendar in our mother document-based  **“**A retrospective cohort study was conducted in 568 under-five children who were admitted for SAM treatment at Pawe General Hospital **from 1^st^ January 2015 up to December 31, 2019.”** | Accepted |
| #3 | The authors should also discuss changes in treatment protocols over time, as updates to the WHO guidelines were released in 2013 for example.  -It would also be important to explain the admission criteria for this treatment center | Operational words | -This is recommendations below were confirmed as current WHO to SAM management of children who are 6–59 months of age  -Weight-for-height ≤–3 Z-score, or  -Mid-upper-arm-circumference<115 mm, or presence of bilateral edema, & failed appetite test were admission criteria  -However, visible severe wasting is not included as a diagnostic criterion. | Accepted & incorporated |
| #4 | In the abstract and the introduction, the authors use the idiom "lion's share" which may not be familiar to all readers, so perhaps this could be changed to more common terminology and to specify whether this is about the absolute number of deaths or mortality in the comparison group | Abstract & Introduction | -We had changed the term “lions share” substituted by phrases “largest proportions” | Accepted and incorporated |
| #5 | : In the abstract, is also unclear what the authors mean by "not take in" the F-75/F-100 formula.  Do the authors mean feeding difficulties or loss of appetite, or that children were not provided these formulas? | Abstract & result part | -We build another model and removed those due to confounding, | Accepted incorporated |
| #6 | The sample size calculation is unclear and appears that it may be appropriate for a prospective study rather than a retrospective analysis and explained. | Sample size | We determined the minimum sample size using a single population proportion formula [n =[(Za/2)2P (1-P)]/d2] by assuming 95% confidence level (Z a/2 = 1.96), margin of error 5% overall SAM mortality rate from previous research46%[6], and 15 % addition for missing/incomplete data. The final sample size was 442. However, when the five years multi-chart was reviewed, 578 records were there and all were considered for analysis*.* |  |
| #7 | The terms kwashiorkor and marasmus should be replaced by edematous malnutrition and severe wasting, respectively | Result part | We accepted and changed terms on result and discussion par.t | Accepted & changed |
| #8 | The authors describe the mean weights of children with SAM, yet it would be more useful to provide z-scores if possible since it is difficult to interpret weights alone. | Result part | -We made, as it is mean weight as it is rather Z-score calculation for all is tedious readers | Not changed |
