# Supplementary material for: Incidence and predictors of severe acute malnutrition mortality in children aged 6–59 months admitted at Pawe general hospital, Northwest Ethiopia
Source: PLoS One. 2022 Feb 25;17(2):e0263236. doi: 10.1371/journal.pone.0263236 (PMC8880861; doi:10.1371/journal.pone.0263236)
Supplement: Attachment — Submitted filename: Response to Reviewers Version 4-5.docx [file pone.0263236.s010.docx]

**Version =6**

**Date = 24 octobers 2021**

**Author’s response to reviews**

**Titles:-INCIDENCE AND PREDICTORS OF SEVERE ACUTE MALNUTRITION MORTALITY RATE AMONG 6-59 MONTHS CHILDREN IN PAWE GENERAL HOSPITAL NORTHWEST ETHIOPIA 2021**

**Authors email**

1) **Fassikaw Kebede:-**[**fassikaw123@gmail.com**](mailto:fassikaw123@gmail.com)
2) **Belete Negese: -** [**yabebalij@gmail.com**](mailto:yabebalij@gmail.com)
3) **Tsehay kebede: - tsehaynolawit@gmail.com**
4) **Atitegeb Abera:-**[**atitegebabera@gmail.com**](mailto:atitegebabera@gmail.com)
5) **Getahun Fentaw:-**[**gechfentaw1014@gmail.com**](mailto:gechfentaw1014@gmail.com)

*DR Walter RJ Taylor Comment*

*Academic editor of* ***[PLoS ONE Journal](https://journals.plos.org/plosone/)***

**EDITORS COMMENT ON PONE-D-21-11027-R-6**

|  | comment | Part | Correction | amendemnet |
| --- | --- | --- | --- | --- |
| #1 | The standard of English needs to be improved and I have been through the paper and made some suggestions | Whole document | Accepted & incorporated |  |
| #2 | I do not understand the national protocol and the 11% - is <11% the target death rate ?  Hopefully, after you include the suggestions, the English will be acceptable. | Abstract | Removed it | Amended |
| #3 | I do not see the value of Table 4 and the multiple Figures when Table 5 contains the key information on the independence of the variables.  Moreover, there are issues with the Figures. | Tables | corrected &  incoprposrted | Amended eache figures |
| #4 | Figure 1 is inconsistent with the reported death rate in the text of 16.03%. From the Figure 1 the cumulative risk of death is about 75%. I suggest you remove Figure 1. | Figures | Accepted and corrected |  |
| #5 | I suggest you recheck all the data in Tables 4 and 5 and the delete all the Figures. Show the data in Table 4 so we can e.g. see the death rates in each category and the p values. There is no need to show the chi squared values as well. | Table | Table-4 is delated but not Tabel-5 | Accepted |

|  | **Comment** | **Part** | **Correction** | **Amendment** |
| --- | --- | --- | --- | --- |
| **#1** | “During SAM inpatient treatment recovery, transfer out, lost follow-up, and death was found 326 (57.39%), 106(18.66%), 46 (8.10%), and 91(16.02%), respectively.  The overall incidence of the mortality rate was 16.03 per 100 (95%CI: 13.86; 20.04) person-days observations  **“*why you put two different rates for mortality”***  ***“Operational words” with Definitions****”* | **Abstract** | ***Accepted and corrected*** | Accepted & amended |
| **#2** | “or mid-upper-arm circumference ≤115 mm, or presence of bilateral edema, and failed appetite test should be admitted for inpatient care[23].The current MUAC definition is <115 mm. Please change. The cited reference is fine. | **Methods** | ***Accepted and corrected*** | Amended  Look Page 6  Reference (23) |
| **#3** | The Sphere cut off is <10% but <11% in the Abstract.  Please correct. | **Abstract** | ***Accepted & incorporated*** | **Accepted**  **Look 2&11** |
| **#4** | A majority usually means > 50% so please amend this sentence. Why not say that mortality in your series was high and half the deaths occurred within 2 weeks. Then list the key factors and suggest clinical care be focused preferentially on these risk factors | **Conclusion** | **Accepted** |  |
| **#5** | *Table 1. I cannot find the drug “Permaquinin” on Google or in the Ethiopian Guidelines for treating SAM. Please tell us the name of this drug.* | **Table 3** | It is cut out from interpretation, But you can read about Primaquine on  ***For further more please read it or brows it***  *https://en.wikipedia.org/wiki/Primaquine* | **Amended** |
| **#6** | Table 2. Please explain what “Waiting time” means. | **Table 2** | ***Interpreted as hospital length of stay during treatment*** | **Amended** |
| **#7** | Table 4. This is unclear. Is it showing an overall survival rate for all the children? If so, 21.09% survival is much less than the reported survival in the paper:“At the end of this study period, 326 (57.39%) admitted under-five children had been cured | **Table 4** | **Removed & corrected and interpreted** | **Amended** |
| **#8** | Figures. Labelling of the Y axes of the 3 figures has been cut away. Please make sure we can see the labels. |  | **It is re interpreted based on your request** | **Accepted and amended** |
| **#9** | Figure 3. This makes no sense to me. It shows a very low survival rate. I suggest you either remove this Figure or put in the correct KM curve. | **Figure 3** | ***Changed based on your queries and re- interpreted*** | **Amended** |
| **#10** | Figure 7. Analysis is not spelt correctly on the X-axis. | **Figure 7** | **Removed and re interpreted what you want to changed** | **Accepted and amended** |
|  |  |  |  |  |

**Reviewer reports:**

| **Reviewer #1:** | | **Area** | **Response of Authors** | **Status** |
| --- | --- | --- | --- | --- |
| #1 | Abstract as grammatical errors | Abstract | We made the extensive grammatical correction and rephrasing with by authors and senior researcher consultation. | Accepted &Amended |
| #2 | Was the study conducted in one or many hospitals author said ‘ hospitals’ | Title &Study area | The study conducted only one hospital i.e. Pawe General Hospital | Fully amended |
| #3 | Provide more details about the Stabilizing Center. Is this award treating both admission illness and nutrition deficiency or for SAM children? | Study area | -After SAM cases diagnosed in the pediatrics ward then transferred to stabilizing center  -which is isolated built for the only treatment of SAM cases and their complications  -Pediatrician and Trained health personnel were used supervised overall SC treatment based on national protocol | Amended %  Incorporated |
| #4 | Understudy population, the study recruited children under management between 2008 and 2012. This is an approximately ten years old cohort. Is there a valid reason for not studying the latest cohort | Study period &  Study population | -In fact, the SAM observation cohort was registration on a computer-based on Ethiopian colander (EC) which is late almost 7 years.  -However, we have changed and edited our mother document based on Gregorian calendar (GC) form;-  -**Marked corrected study periods and study population is as follows.**  **“**A retrospective cohort study was conducted in 568 under-five children who were admitted for SAM treatment at Pawe General Hospital ***from 1^st^ January 2015 up to December 31, 2019***.” | Accepted |
| #5 | Explain clearly the sample size estimation. What is a½ **=0.05?** What were the cases and non-exposed groups? | Samples size | We determined the minimum sample size by Survival sample size by STATA(SE)\14 sample size calculation  The sample size for the second objective was determined using STATA/SE version-14 by considering the following statistical assumptions two-sided significance level (α = 5%), Za/2 = Z value at 95% confidence interval = 1.96, power(**Z_B_)=**80%, and P = % cumulative occurrence of death rate, 1.65 HR. Accordingly, a total of 440 SAM children’s records were recruited.  *Final* Sample size was  (n) =Event = **(Z _a/2_+Z_B_)2 n= e**  P (Event) **θ ^2^p (1-p) p (e)**  *θ* = ln(HR)  HR= e*^θ^* Where ZB = power of the study.  Za/2 = Z value at 95% confidence interval e = event  P (e) = probability of eventP =cumulative mortality rate from previous research *46%[6],* HR= hazard ratio from previous research (AHR) 1.6 [6] | Accepted |
| #6 | Again, the sample size estimated was 596, which is different from the numbers reported so far. | Sample size issue | We already calculated by single population proportion formula of sample size by using listed assumptions The final sample size was calculated as 578; | Amended |
| #7 | The inclusion criteria contradict what is explaining in the study population about the period of recruitment. Here it is mentioned children with SAM treated 2015-2019 were eligible | Inclusion criteria | The study period is changed and the inclusion criteria issue is resolved as following "All SAM admitted & treatment started children from January 1^st^, 2015, up to December 30, 2019, were considered as the study population and included” | Amended |
| #8 | **What** was the discharge criterion from the Stabilizing Center? | Outcome ascertainment | A child reached: weight-for-height/length is ≥–2 Z-score, and they have had no edema for at least 2  weeks, And/or MUAC (Mid-upper-arm circumference) is >115 mm and they have had no edema for at least 2 weeks.- If there are no danger signs for >15 min. | Fully amended |
| #9 | How were the z-scores calculated? | Methodolog | -It is the probability of a score occurring within a standard normal distribution curve: tells how many standard deviations from the mean your score.  -The formula for calculating a z-score is z = (x-μ)/σ, where x is the raw score, μ is the population mean, and σ is the population standard deviation.  -you can use the z-table and the normal distribution graph to give you a visual about how a z-score of 2.0 means “higher than average” | Accepted |
| #10 | -How was the survival analysis conducted?  -How was exposure time defined?  -Which statistical software has used Any testing of non-informative censoring assumption | Data analysis part | -Time-to-event (TTE) data is unique because the outcome of interest is not only whether or not an event occurred, but also when the event occurred.  -There needed 4 main methodological considerations, target event, the time origin, the time scale, and to describe how participants will exit  -Time origin is the point at which follow-up time starts in the case of our study(January 1^st^, 2015-December 30 2019. we used STATA(SE) version 14 all survival analysis and assumption test  - Informative censoring is analogous to non-ignorable missing data, which will bias the analysis.  -There is no definitive way to test whether censoring is non-informative censoring is reasonable.  -In some instances proposed random sub-sample of censored subjects is followed to determine failure times when incomplete information is available about the survival time of SAM cases. | Accepted |
| #11 | Why were 568 children recruited out of the 596 eligible children? | Sample size issue | -We used for analysis 568 samples out of 578, and 10 participant cards were excluded due to treatment outcomes were not registered. | Corrected & incorporated |
| #12 | **“**457(80.46%) of admitted children were new admissions.” What does this mean? | Result part | -Which means ‘457 cases were new admissions for stabilizing center?  -The remaining 111(19.54) cases were relapse SAM cases & Re-admitted twice within | No changed as it is |
| #13 | The authors reported the defaulted and recovered, these are new terms not defined in the methods | Operational words | We made correction and incorporated | Amended |
| #14 | The authors report “307 (54.35%) admitted children had at least one form of comorbidity” does this mean the remaining children were uncomplicated SAM?  Why would uncomplicated SAM be admitted to the hospital? | Result part | Thanks! this is an expression error  -All 568 SAM admitted children had manifold cases diagnosing and responsible for admission  -Of this 307 (54.35%) had more than two concomitant cases diagnosing and admitting for eg,  (commonly SAM with Pneumonia)+ anemia, or diarrhea or vomiting, etc. | Amended |
| #15 | How was anemia defined? | Operational | Anemia:-was defined and classified based on the mean amount of red blood cell or Hgb levels and classified into two ways:- No anemia >11 g/dL, and Anemia≤10.9 g/dl | Amended |
| *#16* | Were the 432 children managed with antimalarial have a positive slide for malaria? | Result report | *Read*  *https://reference.medscape.com/drug/primaquine-342691* | Amended |
| #17 | Provide the length of time (days) that children were on F75 and F100. | Result | SAM inpatient waiting in Stabilizing center was based on three phases of treatments.  **Phase -1=** In a separate room or space  -starter formula for inpatient management  -Only F-75% formula milk used  -Weight is measured & plotted on the chart.  - Standard clinical signs (vomiting, dehydration, cough, respiration, and liver size were measure  - Amount of F-75 to give during Phase 1 is based on the class of weight (Kg).  -took2-6observation with clinical improvement consideration.  -**Transition phase**=  - Use only the [F100 formula](https://motherchildnutrition.org/malnutrition-management/info/feeding-formulas-f75-f100.html)  -Daily surveillance of the child remains the same in Phase 1.  -F-100 milk given are based on the class of weight (Kg)  - Frequency of feeds should normally be 6 per day.  -Routine antibiotics  also continued as necessary  - A maximum of 4-5 days with considerations of clinical improvement of cases  **Phase -2= Use** [F-100](https://motherchildnutrition.org/malnutrition-management/info/feeding-formulas-f75-f100.html) milk  **-**Every morning assessed temperature, vomiting, dehydration, respiration, etc.  -Determined by clinical improvement starting from 2 days of entry----). | Accepted |
| #18 | “In addition, more than half of the 326 (57.51%) and 104 (18.31%) cases were declared as cured and absent” What does absent mean? | Result & operation words | Look result part of Table-2:-  -After admitted and started SAM treatment Within five years cohort -326 (57.38%) cases had been cured, 91(16.73%) cases had died, **46(8.09)** cases- had been transferred out.**105(18.3**%)cases had lost from follow-up (Abscond) were the similar operational definition for Ethiopia FMOH SAM Guidelines.  You can read [**https://motherchildnutrition.org/resources/pdf/mcn-ethiopia-sam-guidelines.pdf**](https://motherchildnutrition.org/resources/pdf/mcn-ethiopia-sam-guidelines.pdf). | Accept and changed |
| #19 | “The overall mortality incidence rate of severe acute malnutrition during the study period was found (IDR=16.73; 95%CI: 13.86--20.04).”Check the 95% CI correct. | Result part | -We repeatedly assessed what you requested to check the result of the incidence rate. Nevertheless, the result is what we had already reported and similar with –  -There is no result deviation both in STATA (SE) version R/14 software and in SPSS version 16. | Amended |
| #20 | “At the end of three weeks (29 days of follow-up) treatment" three weeks have 21days and not 29 days. Were children on follow-up for a fixed 3 weeks? | Result report | We corrected the grammatical errors | Accepted &Amended |
| #21 | -“Thus, within five years of risk observation, 473 children had censored results & gives 505 person-per-day observations (PDOs).”  -What does this mean? You have not explained who and when censoring was done | Result part | -We remove the term person per day of observation(PDOs) expression from the document  incorporated on the mother document  You can look at page 12, from line 8 | Accepted and incorporated |
| #22 | Why are P-values reported as P≤ rather than the standard P<? | Predictors | We corrected and incorporated | Accepted and amended |
| #23 | -What was the rationale for including F-75 and F-100 in the regression models?-It does not make sense to include these in the models | Result part | We authors corrected and incorporated on the final model. | Accepted and amended |
| #24 | -How long did the children spend in the hospital? How long after admission did the deaths occur? Deaths in the first 3 days)and late deaths? | Result part | Maximum of 40 days but in our set up maximum 29 days please let me invited to see (Tables -4 ) Life tables | Amended |

**Part II= reviewer #2**

| Reviewer #2 comments | | Area of comment | Authors response | Status |
| --- | --- | --- | --- | --- |
| #1 | -The introduction focuses on malnutrition broadly as well as SAM specifically, but sometimes it is unclear which of these definitions the authors are referring to.  -It might be beneficial if the authors mainly focus on SAM and the rationale for examining predictors of mortality in children with SAM. | Introduction | -We accepted & rephrasing part of the introduction part. It is attractive now  -Let me invited you to read and check it. | accepted & changed |
| #2 | This analysis used data from 2008 and the end of 2012. (However, later on, the inclusion criteria are stated between 2015-2019 so it is unclear what years were used.)If the admissions were between 2008 and 2012, what are the potential limitations of using data from several years ago, and why were more recent data not considered | Inclusion criteria | Thanks for point of view  -SAM treatment outcome registration in the facility was based on Ethiopian colander (EC)registration  , which is late almost -7 years from G.C.  -However, we have changed and edited all years based on the Gregorian calendar in our mother document-based  **“**A retrospective cohort study was conducted in 568 under-five children who were admitted for SAM treatment at Pawe General Hospital **from 1^st^ January 2015 up to December 31, 2019.”** | Accepted |
| #3 | The authors should also discuss changes in treatment protocols over time, as updates to the WHO guidelines were released in 2013 for example.  -It would also be important to explain the admission criteria for this treatment center | Operational words | -This is recommendations below were confirmed as current WHO to SAM management of children who are 6–59 months of age  -Weight-for-height ≤–3 Z-score, or  -Mid-upper-arm-circumference<115 mm, or presence of bilateral edema, & failed appetite test were admission criteria  -However, visible severe wasting is not included as a diagnostic criterion. | Accepted & incorporated |
| #4 | In the abstract and the introduction, the authors use the idiom "lion's share" which may not be familiar to all readers, so perhaps this could be changed to more common terminology and to specify whether this is about the absolute number of deaths or mortality in the comparison group | Abstract & Introduction | -We had changed the term “lions share” substituted by phrases “largest proportions” | Accepted and incorporated |
| #5 | : In the abstract, is also unclear what the authors mean by "not take in" the F-75/F-100 formula.  Do the authors mean feeding difficulties or loss of appetite, or that children were not provided these formulas? | Abstract | -We build another model and removed those due to confounding, | Accepted incorporated |
| #6 | The sample size calculation is unclear and appears that it may be appropriate for a prospective study rather than a retrospective analysis and explained. | Sample size | We determined the minimum sample size using a single population proportion formula [n =[(Za/2)2P (1-P)]/d2] by assuming 95% confidence level (Z a/2 = 1.96), margin of error 5% overall SAM mortality rate from previous research46%[6], and 15 % addition for missing/incomplete data. The final sample size was 442. However, when the five years multi-chart was reviewed, 578 records were there and all were considered for analysis*.* |  |
| #7 | The terms kwashiorkor and marasmus should be replaced by edematous malnutrition and severe wasting, respectively | Result | We accepted and changed terms on result and discussion par.t | Accepted & changed |
| #8 | The authors describe the mean weights of children with SAM, yet it would be more useful to provide z-scores if possible since it is difficult to interpret weights alone. | Result | -We made, as it is mean weight as it is rather Z-score calculation for all is tedious readers | Not changed |
