# Supplementary material for: Incidence and predictors of severe acute malnutrition mortality in children aged 6–59 months admitted at Pawe general hospital, Northwest Ethiopia
Source: PLoS One. 2022 Feb 25;17(2):e0263236. doi: 10.1371/journal.pone.0263236 (PMC8880861; doi:10.1371/journal.pone.0263236)
Supplement: Attachment — Submitted filename: Response to Reviewers.docx [file pone.0263236.s012.docx]

**Date: 14/1/2022**

**Version: R-8**

**Manuscript ID: PONE-D-21-11027-R8**

**Manuscript titles: INCIDENCE AND PREDICTORS OF SEVERE ACUTE MALNUTRITION MORTALITY RATE AMONG 6-59 MONTHS CHILDREN IN PAWE GENERAL HOSPITAL NORTHWEST ETHIOPIA 2021**

**Number of Authors**

1) **Fassikaw Kebede:-**[**fassikaw123@gmail.com**](mailto:fassikaw123@gmail.com)
2) **Tsehay kebede: - tsehaynolawit@gmail.com**
3) **Belete Negese: -** [**yabebalij@gmail.com**](mailto:yabebalij@gmail.com)
4) **Atitegeb Abera:-**[**atitegebabera@gmail.com**](mailto:atitegebabera@gmail.com)
5) **Getahun Fentaw:-**[**gechfentaw1014@gmail.com**](mailto:gechfentaw1014@gmail.com)

**Part II= Editors’ comments**

| Editors area of comment Journal Requirements | | Part | **Authors response** |
| --- | --- | --- | --- |
| #1 | Please review your reference list to ensure that it is complete and correct. If you have cited papers that have been retracted, please include the rationale for doing so in the manuscript text, or remove these references and replace them with relevant current references. Any changes to the reference list should be mentioned in the rebuttal letter that accompanies your revised manuscript. If you need to cite a retracted article, indicate the article’s retracted status in the References list and also include a citation and full reference for the retraction notice. | Methods- | Thanks for your comment  For the purpose of good information containing on back ground information of the study area, we were changed reference No.19 of the old version articles entitled as “***UNICEF. Situation Analysis of Children and Women:Beni shangul-Gumuz Region. REPORT 2019”*** by  “**Time to Develop and Predictors for Incidence of Tuberculosis among Children Receiving Antiretroviral Therapy; Tuberculosis Research and Treatment 2021, 2021:6686019.”** Kebede F, Kebede T, Kebede B, Abate A, Jara D, Negese B, Shawano T.  However by the request of acadmic editore ways of new papers cited or retracted have been forbiden as a rules of PLOS ONE Journal Requirements  We have been corrected to be the first article on citation at reference No.19”  In short we have maintiend previouse citated article on the paper emetoled as “***UNICEF. Situation Analysis of Children and Women:Beni shangul-Gumuz Region. REPORT 2019”***    **Please look at reference No-19** |
